# CD57^+^ Memory T Cells Proliferate *In Vivo*

**DOI:** 10.1016/j.celrep.2020.108501

**Published:** 2020-12-15

**Authors:** Raya Ahmed, Kelly L. Miners, Julio Lahoz-Beneytez, Rhiannon E. Jones, Laureline Roger, Christina Baboonian, Yan Zhang, Eddie C.Y. Wang, Marc K. Hellerstein, Joseph M. McCune, Duncan M. Baird, David A. Price, Derek C. Macallan, Becca Asquith, Kristin Ladell

**Affiliations:** 1 Institute for Infection and Immunity, St. George’s, University of London, London SW17 0RE, UK; 2 Division of Infection and Immunity, Cardiff University School of Medicine, Heath Park, Cardiff CF14 4XN, UK; 3 Department of Infectious Disease, Imperial College London, London W2 1PG, UK; 4 Division of Cancer and Genetics, Cardiff University School of Medicine, Heath Park, Cardiff CF14 4XN, UK; 5 Department of Nutritional Sciences and Toxicology, University of California, Berkeley, CA 94720, USA; 6 HIV Frontiers Program, Global Health Innovative Technology Solutions, Bill & Melinda Gates Foundation, Seattle, WA 98109, USA; 7 Systems Immunity Research Institute, Cardiff University School of Medicine, Heath Park, Cardiff CF14 4XN, UK; 8 St George’s University Hospitals NHS Foundation Trust, London SW17 0QT, UK; 9 Neonatal Unit, Singleton Hospital, Swansea Bay University Health Board, Swansea SA2 8QA, UK

**Keywords:** Enter keywords here

## Abstract

A central paradigm in the field of lymphocyte biology asserts that replicatively senescent memory T cells express the carbohydrate epitope CD57. These cells nonetheless accumulate with age and expand numerically in response to persistent antigenic stimulation. Here, we use *in vivo* deuterium labeling and *ex vivo* analyses of telomere length, telomerase activity, and intracellular expression of the cell-cycle marker Ki67 to distinguish between two non-exclusive scenarios: (1) CD57^+^ memory T cells do not proliferate and instead arise via phenotypic transition from the CD57^−^ memory T cell pool; and/or (2) CD57^+^ memory T cells self-renew via intracompartmental proliferation. Our results provide compelling evidence in favor of the latter scenario and further suggest in conjunction with mathematical modeling that self-renewal is by far the most abundant source of newly generated CD57^+^ memory T cells. Immunological memory therefore appears to be intrinsically sustainable among highly differentiated subsets of T cells that express CD57.

## Highlights

• CD57^+^ memory T cells are not replicatively senescent *in vivo*
• CD57^+^ memory T cells are maintained primarily via self-renewal *in vivo*

## Graphical Abstract

**Figure:**
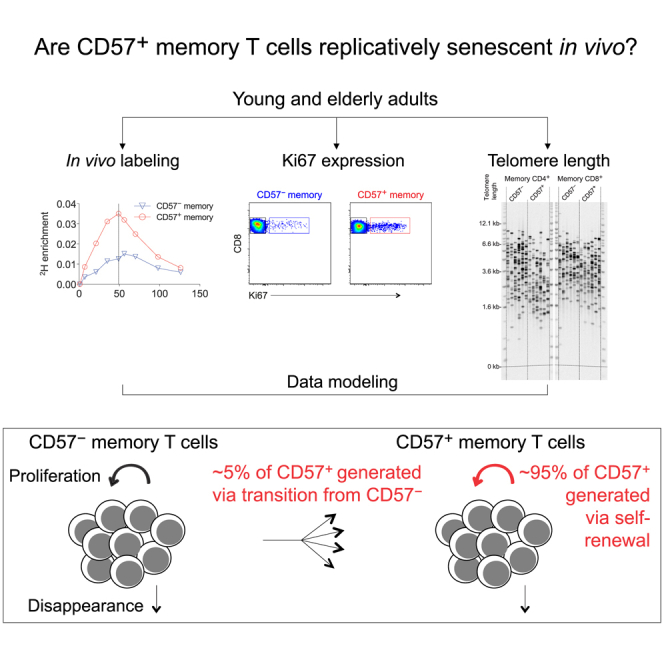

## Teaser

*In vitro* studies have led to the widely held assumption that replicatively senescent memory T cells express the carbohydrate epitope CD57. Using a variety of experimental approaches and mathematical modeling of composite datasets, Ahmed et al. show that CD57^+^ memory T cells proliferate and self-renew *in vivo*.

## Introduction

Immune senescence has been linked with the accumulation of terminally differentiated lymphocytes that fail to proliferate in response to antigenic challenge. It has also been suggested that surface expression of CD57, a terminally sulfated glycan carbohydrate epitope (Abo and Balch, 1981), identifies memory T cells that lack the capacity to proliferate (Brenchley et al., 2003). In line with these widely accepted paradigms, highly differentiated effector memory T cells that express CD45RA, known as T_EMRA_ cells, become more prevalent with age (Nociari et al., 1999) and often express CD57 (Ladell et al., 2008).

Contrary to the notion of differentiation-linked senescence, memory T cells that express CD57 can be induced to proliferate *in vitro*, at least under optimized conditions (Chong et al., 2008; Izquierdo et al., 1990). Parallel strands of evidence have further suggested a key role for these cells as immune effectors. For example, memory CD8^+^ T cells rarely express CD57 in conjunction with programmed death-1 (PD-1) (Petrovas et al., 2009), a marker associated with exhaustion (Day et al., 2006; Freeman et al., 2006; Petrovas et al., 2006; Trautmann et al., 2006), and functionally replete memory CD4^+^ and CD8^+^ T cells with cytotoxic potential typically express high levels of CD57 (Casazza et al., 2006; Chattopadhyay et al., 2009; Chong et al., 2008; Kern et al., 1999; Le Priol et al., 2006; Takata and Takiguchi, 2006; van Leeuwen et al., 2002). Virus-specific memory T cells have also been identified in the T_EMRA_ compartment (Appay et al., 2008). In the CD8^+^ lineage, these cells have been associated with protective effects, most notably during acute (Northfield et al., 2007) and chronic human immunodeficiency virus type 1 (HIV-1) infection (Addo et al., 2007), and in a study of elite controllers with eventual disease progression, increasing levels of viral replication appeared to drive the formation of CD28^−^CD57^+^ memory CD8^+^ T cells, potentially indicating a reactive escalation in the cytotoxic response to HIV-1 (Benito et al., 2018). Accordingly, CD57^+^ memory T cells enrich the immune system with important antigen-dependent effector functions and, by extension, do not necessarily represent an irrelevant “cul-de-sac” in the lymphocyte differentiation pathway.

In this study, we used deuterium labeling to quantify the proliferation of CD57^−^ and CD57^+^ memory T cells *in vivo* and supplemented these analyses with *ex vivo* measurements of telomere length, telomerase activity, and intracellular expression of the cell-cycle marker Ki67. We then used mathematical modeling to evaluate two non-exclusive hypothetical scenarios: (1) CD57^+^ memory T cells arise from the CD57^−^ memory T cell compartment as a consequence of progressive differentiation; and/or (2) CD57^+^ memory T cells self-renew via intracompartmental proliferation and thereby contribute to long-term immunological memory.

## Results

### CD57^−^ and CD57^+^ Memory T Cells Exhibit Similar Rates of Deuterium Incorporation

**Figure 1:**
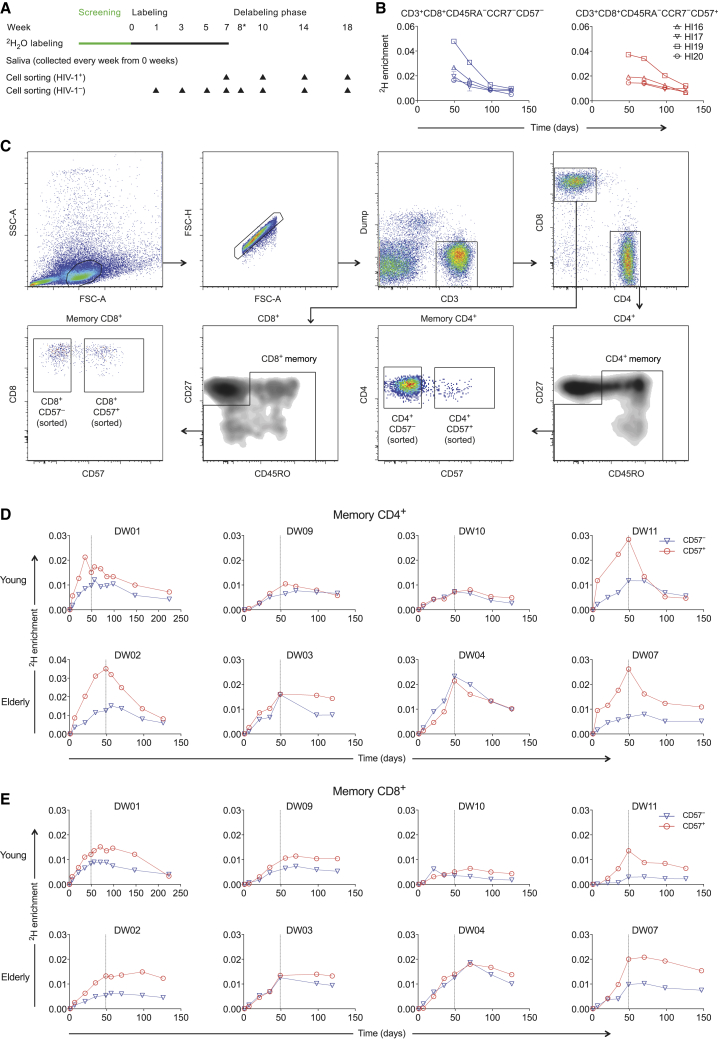
CD57^−^ and CD57^+^ Memory T Cells Exhibit Similar Rates of Deuterium Incorporation (A) Schematic representation of the ^2^H_2_O labeling protocol and sampling time points. (B) Experimental labeling data for CD57^−^ and CD57^+^ memory CD8^+^ T cells sampled from the HIV-1-infected volunteers in cohort 1. The corresponding flow cytometric gating strategy is shown in Figure S1. (C) Successive panels depict the flow cytometric gating strategy used to sort CD57^−^ and CD57^+^ memory T cells from the CD4^+^ and CD8^+^ lineages (cohort 2). Lymphocytes were identified in a forward scatter-area versus side scatter-area plot, and single cells were identified in a forward scatter-area versus forward scatter-height plot. Boolean gates were drawn for analysis only to exclude fluorochrome aggregates. Viable CD3^+^CD14^−^CD19^−^ cells were then identified in the CD4^+^ and CD8^+^ lineages, and sort gates were fixed on CD57^−^ and CD57^+^ memory cells after exclusion of potentially naive CD27^bright^CD45RO^−^ cells. (D) Experimental labeling data for CD57^−^ and CD57^+^ memory CD4^+^ T cells sampled from the healthy volunteers in cohort 2. (E) Experimental labeling data for CD57^−^ and CD57^+^ memory CD8^+^ T cells sampled from the healthy volunteers in cohort 2.

Preliminary *in vivo* labeling data were derived from studies of volunteers with chronic HIV-1 infection (aged 36–53 years), all of whom were antiretroviral drug-free at the time of experimentation and seropositive for cytomegalovirus (CMV; n = 4; Table S1). The labeling protocol is outlined in Figure 1A. Venous blood was sampled at weeks 7 (end of labeling), 10, 14, and 18, and at each time point, CD57^−^ and CD57^+^ memory CD8^+^ T cells were flow-sorted from the CD45RA^−^CCR7^−^ subset at >98% purity (Figure S1). This gating strategy was designed to exclude T_EMRA_ cells, which were assessed separately in an earlier report (Ladell et al., 2008). Considerable rates of ^2^H labeling and delabeling were observed among CD45RA^−^CCR7^−^CD57^−^ and CD45RA^−^CCR7^−^CD57^+^ memory CD8^+^ T cells (Figure 1B).

Immune activation enhances the turnover of memory T cells in the setting of chronic HIV-1 or HIV-2 infection (Hegedus et al., 2014; McCune et al., 2000; Vrisekoop et al., 2015; Zhang et al., 2013). We therefore sought to confirm these preliminary findings in a more comprehensive labeling study of healthy volunteers (aged 29–83 years), all of whom were seronegative for HIV-1 and seropositive for CMV. Recruitment was stratified to include equal numbers of young (aged 29–47 years) and elderly individuals (aged 60–83 years), the latter representing a population in which immune senescence was more likely (total n = 8; Table S1). Venous blood was sampled during the labeling phase (weeks 1, 3, and 5), at the end of labeling (week 7), and during the delabeling phase (weeks 8, 10, 14, and 18) (Figure 1A). At each time point, CD57^−^ and CD57^+^ memory T cells were flow-sorted from the CD4^+^ and CD8^+^ lineages at >98% purity after gating out potentially naive CD27^bright^CD45RO^−^ events (Figures 1C and S1).

In each coreceptor-defined lineage, similar patterns of ^2^H labeling and delabeling were observed among CD57^−^ and CD57^+^ memory T cells, and equivalent (n = 3) or greater rates of ^2^H labeling (n = 5) were observed among CD57^+^ memory T cells compared with CD57^−^ memory T cells (Figures 1D and 1E). Importantly, the corresponding ^2^H label enrichments in body water followed an expected rise-and-fall profile (Figure S2), and in the context of age-related immune senescence, no intralineage or intrasubset differences in the kinetics of ^2^H accumulation or loss were apparent between young and elderly volunteers (Figures 1D and 1E).

### Ki67^+^ Cells Are Readily Detectable in the CD57^+^ Memory T Cell Pool

**Figure 2:**
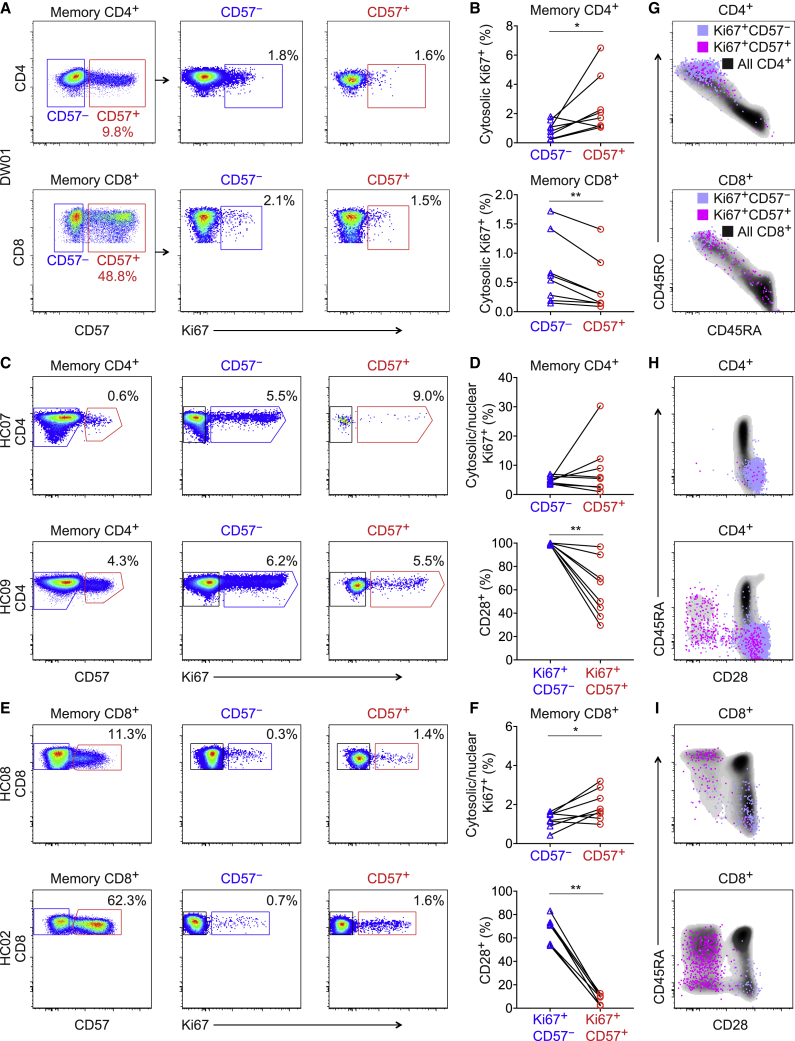
Ki67^+^ Cells Are Readily Detectable in the CD57^+^ Memory T Cell Pool (A) Representative flow cytometric data from a labeled volunteer (DW01) showing cytosolic expression of Ki67 among memory CD4^+^ (top) or CD8^+^ T cells (bottom) gated as CD57^−^ (blue) or CD57^+^ (red). (B) Percent cytosolic expression of Ki67 among memory CD4^+^ (top) or CD8^+^ T cells (bottom) gated as CD57^−^ (blue triangles) or CD57^+^ (red circles). ^∗^p < 0.05, ^∗∗^p < 0.01. Paired samples Wilcoxon test. (C) Representative flow cytometric data from unlabeled volunteers (n = 2) showing cytosolic/nuclear expression of Ki67 among memory CD4^+^ T cells gated as CD57^−^ (blue) or CD57^+^ (red). HC07 was seronegative for CMV. (D) Top: percent cytosolic/nuclear expression of Ki67 among memory CD4^+^ T cells gated as CD57^−^ (blue triangles) or CD57^+^ (red circles). Bottom: percent expression of CD28 among the corresponding Ki67^+^CD57^−^ (blue triangles) and Ki67^+^CD57^+^ memory CD4^+^ T cells (red circles). ^∗∗^p < 0.01. Paired samples Wilcoxon test. (E) Representative flow cytometric data from unlabeled volunteers (n = 2) showing cytosolic/nuclear expression of Ki67 among memory CD8^+^ T cells gated as CD57^−^ (blue) or CD57^+^ (red). HC02 was seropositive for CMV, and HC08 was seronegative for CMV. (F) Top: percent cytosolic/nuclear expression of Ki67 among memory CD8^+^ T cells gated as CD57^−^ (blue triangles) or CD57^+^ (red circles). Bottom: percent expression of CD28 among the corresponding Ki67^+^CD57^−^ (blue triangles) and Ki67^+^CD57^+^ memory CD8^+^ T cells (red circles). ^∗^p < 0.05, ^∗∗^p < 0.01. Paired samples Wilcoxon test. (G) Phenotypic characteristics of Ki67^+^CD57^−^ and Ki67^+^CD57^+^ memory CD4^+^ (top) or CD8^+^ T cells (bottom) shown overlaid on density clouds representing the corresponding total CD4^+^ (top) or CD8^+^ T cell populations (bottom). Related to (A). (H) Phenotypic characteristics of Ki67^+^CD57^−^ and Ki67^+^CD57^+^ memory CD4^+^ T cells shown overlaid on density clouds representing the corresponding total CD4^+^ T cell populations. Related to (C). Key as in (G). (I) Phenotypic characteristics of Ki67^+^CD57^−^ and Ki67^+^CD57^+^ memory CD8^+^ T cells shown overlaid on density clouds representing the corresponding total CD8^+^ T cell populations. Related to (E). Key as in (G).

To corroborate these findings, we measured the expression of Ki67, an intracellular marker that accumulates during active phases of the cell cycle (Gerdes et al., 1983; Miller et al., 2018). Cytosolic expression of Ki67 was detected in the CD4^+^ lineage at mean frequencies of 1% among CD57^−^ memory T cells and 2.9% among CD57^+^ memory T cells (p = 0.02, paired samples Wilcoxon test; Figures 2A and 2B) and in the CD8^+^ lineage at mean frequencies of 0.7% among CD57^−^ memory T cells and 0.4% among CD57^+^ memory T cells (p = 0.008, paired samples Wilcoxon test; Figures 2A and 2B). Higher frequencies were observed using a different approach that simultaneously exposed intranuclear antigens. Cytosolic/nuclear expression of Ki67 was detected in the CD4^+^ lineage at mean frequencies of 4.9% among CD57^−^ memory T cells and 8.6% among CD57^+^ memory T cells (p = 0.742, paired samples Wilcoxon test; Figures 2C and 2D) and in the CD8^+^ lineage at mean frequencies of 1.2% among CD57^−^ memory T cells and 1.9% among CD57^+^ memory T cells (p = 0.039, paired samples Wilcoxon test; Figures 2E and 2F).

In further analyses, we assessed the phenotypic characteristics of Ki67^+^CD57^−^ and Ki67^+^CD57^+^ memory T cells in the CD4^+^ and CD8^+^ lineages. As expected, Ki67^+^ memory CD4^+^ T cells predominantly expressed CD45RO, with or without CD57, whereas Ki67^+^ memory CD8^+^ T cells were phenotypically more heterogeneous and often expressed CD45RA in conjunction with CD57 (Figure 2G). Moreover, Ki67^+^CD57^−^ memory T cells expressed CD28 at higher frequencies than Ki67^+^CD57^+^ memory T cells, both in the CD4^+^ lineage (p = 0.008, paired samples Wilcoxon test; Figures 2D and 2H) and in the CD8^+^ lineage (p = 0.008, paired samples Wilcoxon test; Figures 2F and 2I). Similar patterns of expression were observed for CCR7 (Figure S3).

To link these findings with the labeling data, we compared the phenotypic characteristics of Ki67^+^CD57^−^ and Ki67^+^CD57^+^ memory T cells with the phenotypic characteristics of CD57^−^ and CD57^+^ memory T cells sampled from the healthy volunteers in cohort 2. In both coreceptor-defined lineages, CD57^−^ memory T cells expressed CD28 and CCR7 at higher frequencies than CD57^+^ memory T cells, akin to the corresponding Ki67^+^ memory T cells (Figure S4). Of note, CD57^+^ memory CD4^+^ T cells mostly lacked CD27 but commonly expressed CD127 and PD-1, whereas CD57^+^ memory CD8^+^ T cells were generally more differentiated and rarely expressed CD27, CD127, or PD-1 (Figures S4 and S5). Age had no apparent influence on these phenotypic characteristics (data not shown).

### CD57^−^ and CD57^+^ Memory T Cells Have Similar Division Histories

**Figure 3:**
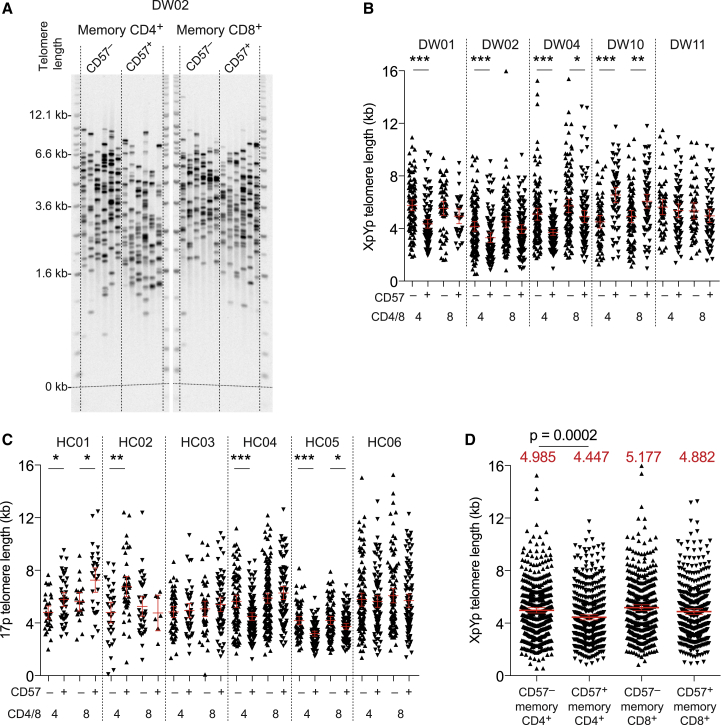
CD57^−^ and CD57^+^ Memory T Cells Have Similar Division Histories (A) Representative single telomere length analysis (STELA) data showing XpYp telomere lengths among CD57^−^ and CD57^+^ memory CD4^+^ or CD8^+^ T cells sampled from a labeled volunteer (DW02). (B) XpYp telomere lengths among CD57^−^ and CD57^+^ memory CD4^+^ or CD8^+^ T cells sampled from labeled volunteers (cohort 2). ^∗^p < 0.05, ^∗∗^p < 0.01, ^∗∗∗^p < 0.001. Mann-Whitney *U* test. (C) 17p telomere lengths among CD57^−^ and CD57^+^ memory CD4^+^ or CD8^+^ T cells sampled from unlabeled volunteers (cohort 3). ^∗^p < 0.05, ^∗∗^p < 0.01, ^∗∗∗^p < 0.001. Mann-Whitney *U* test. (D) Pooled XpYp telomere length data for the volunteers shown in (B). Red lines show means with 95% confidence intervals. Mean values are specified above each column. Significance was assessed using the Mann-Whitney *U* test.

To refine our understanding of these datasets, we measured XpYp or 17p telomere lengths in the CD57^−^ and CD57^+^ memory T cell pools (Figure 3A). Telomere lengths were distributed in a heterogeneous manner and overlapped considerably across CD57-defined subsets in the CD4^+^ and CD8^+^ lineages. In some volunteers, significant differences in mean telomere length were observed between the CD57^−^ and CD57^+^ memory T cell populations, most commonly in the CD4^+^ lineage, but no consistent directional change was apparent between CD57-defined subsets in either the CD4^+^ or the CD8^+^ lineage (Figures 3B and 3C). However, pooling the XpYp data from labeled volunteers revealed that telomere lengths were maintained to a slightly greater extent in the CD57^−^ memory CD4^+^ T cell population compared with the CD57^+^ memory CD4^+^ T cell population (Figure 3D), and pooling the 17p data from volunteers in cohort 3 yielded a similar result with borderline significance (p = 0.037, Mann-Whitney *U* test; data not shown). Telomerase activity was generally low, as expected given the infrequent expression of Ki67, but marginally higher levels were detected among CD57^−^ memory T cells compared with CD57^+^ memory T cells in both coreceptor-defined lineages. These data were reported previously for reference in another labeling study of the volunteers in cohort 2 (Ahmed et al., 2016).

### CD57^−^ and CD57^+^ Memory T Cells Self-Renew *In Vivo*

**Figure 4:**
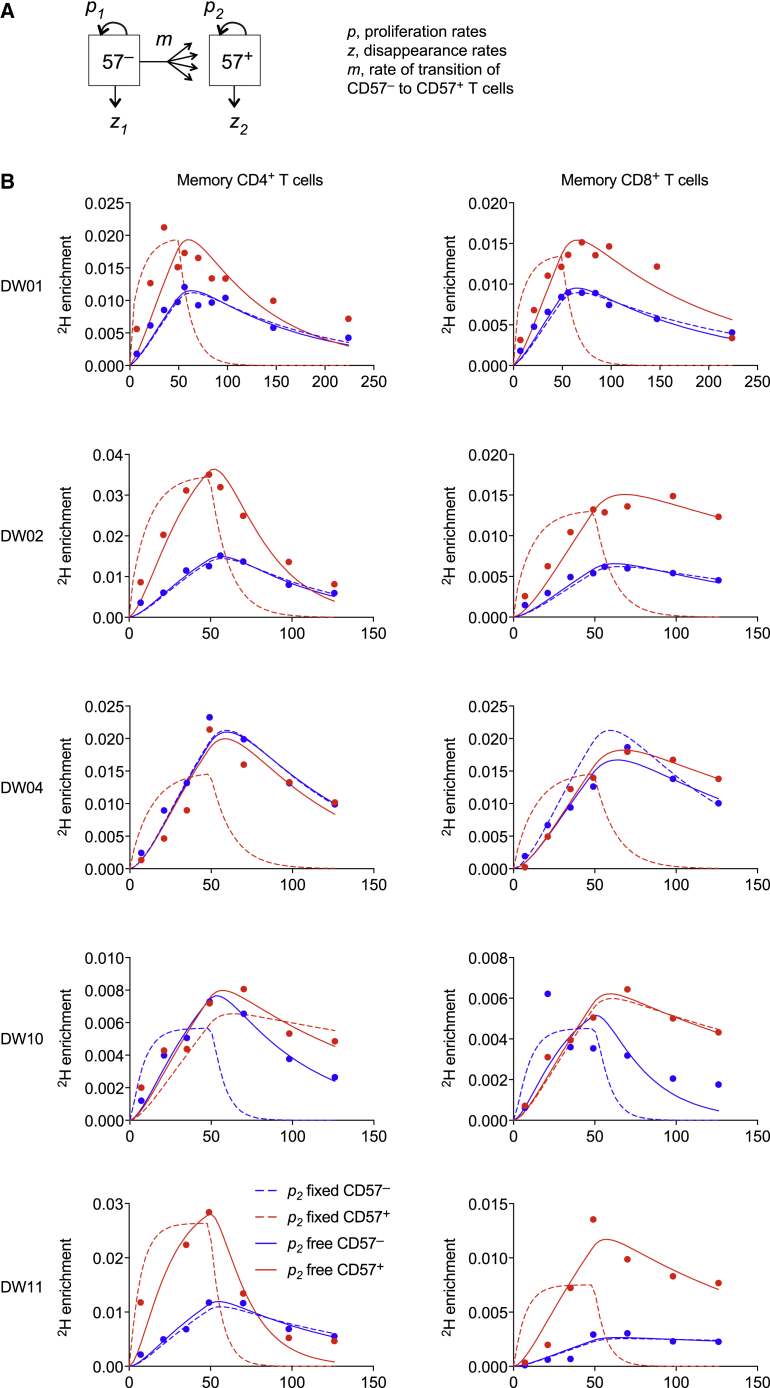
CD57^−^ and CD57^+^ Memory T Cells Self-Renew *In Vivo* (A) Schematic representation of the mathematical model. (B) Model fits to the measured data (dots) for CD57^−^ and CD57^+^ memory CD4^+^ (left) or CD8^+^ T cells (right) with *p*_2_ constrained to zero (dashed lines) or free (solid lines).

To integrate these findings, we fitted mathematical models simultaneously to the ^2^H enrichment data and the telomere length data, allowing CD57^−^ memory T cells to become CD57^+^ memory T cells in the CD4^+^ and CD8^+^ lineages (Figure 4A). This approach was designed to capture both possible explanations for the accumulation of label in the corresponding CD57^+^ compartments, namely that CD57^−^ memory T cells proliferated and acquired expression of CD57 and/or that CD57^+^ memory T cells proliferated and retained expression of CD57. Proliferation rates were denoted by *p*_1_ and *p*_2_ for CD57^−^ and CD57^+^ memory T cells, respectively, such that replicative senescence in the CD57^+^ subsets was represented by the constraint *p*_2_ = 0. Telomeres shorten by an average of 50 bp per cell division (De Boer and Noest, 1998). In some cases, this rate of erosion can be counteracted by the activity of telomerase, a possibility that was included in the model assumptions via an additional parameter, termed *K*.

Fits were restricted to the volunteers for whom labeling data and telomere length data were available (n = 5). The general model (with *p*_2_ free) fitted the data well for CD57^−^ and CD57^+^ memory T cells in the CD4^+^ and CD8^+^ lineages (Figure 4B; Table 1). Importantly, the proliferation rate estimates for the CD57^+^ subsets were positive, with 95% confidence intervals that did not overlap zero (i.e., *p*_2_ > 0). This conclusion was robust to different rates of telomere shortening per cell division and changes in the parameter *K* (Figure S6). Model performance was considerably worse if proliferation was disallowed in the CD57^+^ memory T cell populations (i.e., *p*_2_ = 0) (Figures 4B and S7). Indeed, the median p value in a comparison of the models was 5 × 10^−20^ (F test), which provided strong evidence to reject the null hypothesis of the simpler model, namely that CD57^+^ memory T cells were unable to proliferate (i.e., *p*_2_ = 0). Moreover, the median small-sample-corrected Akaike information criterion (AICc) difference was 106, which indicated that the simpler model provided a substantially worse description of the data (i.e., fit after adjustment for model complexity). The best fits were therefore consistent with substantial proliferation in the CD57^+^ compartments, such that influx from the CD57^−^ compartments typically contributed only ∼5% of all newly generated CD57^+^ memory T cells (Table 1).

**Table 1:** Parameter Estimates from Model Fits to the Experimental Data

ID	*p*_1_ (% per day)	SE (% per day)	*z*_1_ (% per day)	SE (% per day)	*p*_2_ (% per day)	SE (% per day)	*z*_2_ (% per day)	SE (% per day)	*mR* (% per day)	SE (% per day)	Self-renewal (%)
CD4^+^ T Cells											
DW01	0.33	0.04	0.0088	0.27	0.59	0.08	1.24	0.37	0.02	0.01	97
DW02	0.65	0.06	1.89	0.31	2.08	0.23	3.77	0.58	0.17	0.03	93
DW04	0.53	N/D	1.43	N/D	0.53	N/D	1.65	N/D	0.00	N/D	100
DW10	0.58	0.05	1.80	0.28	0.49	0.05	0.92	0.22	0.00	0.00	99
DW11	0.44	0.06	1.24	0.33	1.64	0.02	5.24	0.66	0.45	0.02	79
Median	0.53		1.43		0.59		1.65		0.02		97
CD8^+^ T Cells											
DW01	0.26	0.02	0.73	0.13	0.40	0.03	0.70	0.13	0.02	0.00	96
DW02	0.22	0.02	0.86	0.22	0.42	0.04	0.45	0.19	0.04	0.01	92
DW04	0.37	N/D	0.91	N/D	0.37	N/D	0.62	N/D	0.00	N/D	100
DW10	0.55	0.17	3.60	1.53	0.34	0.11	0.63	0.56	0.02	0.02	95
DW11	0.07	0.01	0.24	0.34	0.32	0.03	0.81	0.04	0.07	0.01	83
Median	0.26		0.86		0.37		0.63		0.02		95

The best-fit estimates are shown. A limited number of data points were available from one volunteer (DW04). The asymptotic covariance matrix method was used to calculate standard errors (SEs). The percentage of new CD57^+^ T cells generated via intracompartmental proliferation (right column) was calculated as 100 × *p*_2_/(*p*_2_ + *mR*). ID, identification number; N/D, not determined.

## Discussion

In this study, we used *in vivo* deuterium labeling and *ex vivo* analyses of telomere length, telomerase activity, and intracellular expression of the cell-cycle marker Ki67 to investigate the paradigm that replicatively senescent memory T cells can be identified via the surrogate marker CD57. We detected similar rates of proliferation among CD57^−^ and CD57^+^ memory T cells in both coreceptor-defined lineages. These results were supported by flow cytometric analyses, which revealed the presence of actively dividing cells in the corresponding CD57^+^ memory T cell populations. Marginally higher levels of telomerase activity were detected among CD57^−^ memory T cells compared with CD57^+^ memory T cells in the CD4^+^ and CD8^+^ lineages, consistent with a relatively small biological effect, and in line with recent observations (Fali et al., 2018), telomere lengths were maintained to a slightly greater extent among CD57^−^ memory CD4^+^ T cells compared with CD57^+^ memory CD4^+^ T cells. In contrast, telomere lengths were distributed around similar means in the CD57^−^ and CD57^+^ memory CD8^+^ T cell populations. Mathematical modeling of the experimental data further suggested that self-renewal via intracompartmental proliferation rather than replenishment via phenotypic conversion was by far the most abundant source of newly generated CD57^+^ memory CD4^+^ and CD57^+^ memory CD8^+^ T cells.

CD57 was originally recognized as a differentiation antigen on the surface of NK cells (Abo and Balch, 1981) and subsequently associated with other lymphocyte subsets in germinal centers (Ritchie et al., 1983). In peripheral blood, CD57^+^ memory T cells accumulate throughout life, especially after infection with CMV (Gratama et al., 1989). These associations with age and persistent antigenic drive were mechanistically linked in a seminal *in vitro* study, which reported that replicatively senescent memory CD8^+^ T cells expressed CD57 (Brenchley et al., 2003). However, an earlier study had reached a different conclusion (Izquierdo et al., 1990), and later experiments showed that CD57^+^ memory CD8^+^ T cells were able to proliferate *in vitro* in the presence of certain growth factors, potentially mimicking the *in vivo* microenvironment (Chong et al., 2008). Similar findings were reported in another study, although markedly higher response frequencies on a per-cell basis were noted in the CD57^−^ subset compared with the CD57^+^ subset (Le Priol et al., 2006). Nonetheless, the proportion of responding cells in the CD57^+^ subset was more than sufficient to maintain homeostatic turnover, at least according to a deuterium labeling study of bulk memory T cell populations (Zhang et al., 2013).

T_EMRA_ cells are somewhat resistant to apoptosis (Gupta and Gollapudi, 2007) and retain deuterium in the CD8^+^ lineage with an estimated half-life of approximately 25 years, assuming simple exponential decay without phenotypic conversion (Ladell et al., 2008). In response to extreme stimulation with supraphysiological concentrations of phytohemagglutinin and interleukin-2, CD8^+^ T_EMRA_ cells that expressed CD57 were recently found to be more susceptible to cell death than CD8^+^ T_EMRA_ cells that lacked CD57 (Verma et al., 2017). This observation was thought to indicate a functional dichotomy between CD57-defined subsets within the CD8^+^ T_EMRA_ compartment. However, it does not necessarily follow that a similar dichotomy exists under homeostatic conditions, because terminally differentiated CD57^+^ memory CD8^+^ T cells may be protected from excessive stimulation *in vivo* by a lack of costimulatory receptors, such as CD27 and CD28.

In summary, we have shown that CD57^+^ memory T cells in the CD4^+^ and CD8^+^ lineages self-renew *in vivo*, enabling the long-term maintenance of functionally replete immunological memory. It remains to be determined how this process is regulated in terms of antigenic drive versus homeostatic signals as a function of differentiation status, but nonetheless, it is clear from the presented data that replicatively senescent memory T cells cannot be defined solely via surface expression of CD57.

## STAR★Methods

### Key Resources Table

**Table:** 

REAGENT or RESOURCE	SOURCE	IDENTIFIER
**Antibodies**		

Anti-CD3–APC-H7 (clone SK7)	BD Biosciences	Cat#641415; RRID:AB_2870309
Anti-CD4–PE-Cy5.5 (clone S3.5)	Thermo Fisher Scientific	Cat#MHCD0418; RRID:AB_10376013
Anti-CD8–BV711 (clone RPA-T8)	BioLegend	Cat#301044; RRID:AB_2562906
Anti-CD14–V500 (clone M5E2)	BD Biosciences	Cat#561391; RRID:AB_10611856
Anti-CD19–V500 (clone HIB19)	BD Biosciences	Cat#561121; RRID:AB_10562391
Anti-CD27–QD605 (clone CLB-27/1)	Thermo Fisher Scientific	Cat#Q10065; RRID:AB_2556450
Anti-CD28–APC (clone CD28.2)	BD Biosciences	Cat#559770; RRID:AB_398666
Anti-CD28–BV421 (clone CD28.2)	BioLegend	Cat#302930; RRID:AB_2561910
Anti-CD45RA–ECD (clone 2H4LDH11LDB9)	Beckman Coulter	Cat#M2711U; RRID:AB_10640553
Anti-CD45RA–PE (clone HI100)	BD Biosciences	Cat#555489; RRID:AB_395880
Anti-CD45RO–ECD (clone UCHL1)	Beckman Coulter	Cat#IM2712U; RRID:AB_10639537
Anti-CD57–FITC (clone NK-1)	BD Biosciences	Cat#555619; RRID:AB_395986
Anti-CD57–PE-Cy7 (clone NK-1)	BioLegend	Cat#359624; RRID:AB_2632689
Anti-CD127–BV421 (clone A019D5)	BioLegend	Cat#351310; RRID:AB_10960140
Anti-CCR7–BV421 (clone G043H7)	BioLegend	Cat#353208; RRID:AB_11203894
Anti-CCR7–FITC (clone 150503)	BD Biosciences	Cat#561271; RRID:AB_10561679
Anti-CCR7–PE-Cy7 (clone 3D12)	BD Biosciences	Cat#557648; RRID:AB_396765
Anti-CXCR3–BV421 (clone G025H7)	BioLegend	Cat#353716; RRID:AB_2561448
Anti-Ki67–AF647 (clone B56)	BD Biosciences	Cat#558615; RRID:AB_647130
Anti-Ki67–FITC (clone B56)	BD Biosciences	Cat#556026; RRID:AB_396302
Anti-PD-1–BV421 (clone EH12.2H7)	BioLegend	Cat#329920; RRID:AB_10960742

**Biological Samples**		

Peripheral blood from adults infected with HIV-1	San Francisco General Hospital, San Francisco, CA, USA	N/A
Peripheral blood from healthy adult volunteers	St George’s Hospital, London, UK	N/A
Peripheral blood from healthy adult volunteers	Cardiff University School of Medicine, Cardiff, UK	N/A

**Chemicals, Peptides, and Recombinant Proteins**		

Heavy water (^2^H_2_O)	Cambridge Isotope Laboratories	Cat#DLM-4TPB-PK
Pentafluorobenzyl hydroxylamine	Sigma-Aldrich	Cat#194484
Sodium dodecyl sulfate	Thermo Fisher Scientific	Cat#10593355
Tris(hydroxymethyl) methylamine	Thermo Fisher Scientific	Cat#77-86-1
Ethylenediaminetetraacetic acid	Sigma-Aldrich	Cat#E6511-100G
Sodium hydroxide	Thermo Fisher Scientific	Cat#J/7620/15
Sodium phosphate dibasic	Sigma-Aldrich	Cat#7558-79-4
Hydrochloric acid	Thermo Fisher Scientific	Cat#7647-01-0

**Critical Commercial Assays**		

LIVE/DEAD Fixable Aqua Dead Cell Stain Kit	Thermo Fisher Scientific	Cat#L34966
Cytofix/Cytoperm Kit	BD Biosciences	Cat#554715
Foxp3 Transcription Factor Staining Buffer Kit	Thermo Fisher Scientific	Cat#00-5521-00
QIAmp DNA Mini Kit	QIAGEN	Cat#51304
QIAmp DNA Micro Kit	QIAGEN	Cat#56304

**Oligonucleotides**		

XpYpE2: TTGTCTCAGGGTCCTAGTG	Eurofins Genomics	Custom
17pserev1: GAATCCACGGATTGCTTTGTGTAC	Eurofins Genomics	Custom
Telorette2: TGCTCCGTGCATCTGGCATCTAACCCT	Eurofins Genomics	Custom
Teltail: TGCTCCGTGCATCTGGCATC	Eurofins Genomics	Custom

**Software and Algorithms**		

DiVa version 8	BD Biosciences	https://www.bdbiosciences.com/en-us
FlowJo software version 9.9.4	FlowJo LLC	https://www.flowjo.com
Phoretix 1D Quantifier	Nonlinear Dynamics	http://www.nonlinear.com/about/totallab
Prism version 8	GraphPad	https://www.graphpad.com

**Other**		

DreamTaq polymerase	Thermo Fisher Scientific	Cat#EP0702
Pwo polymerase	Sigma-Aldrich	Cat#11644955001
dNTPs	Promega	Cat#U1511
α-^33^P dCTP	PerkinElmer	Cat#BLU013H100UC
Megaprime	VWR	Cat#RPN1607
Hybond-XL	VWR	Cat#RPN15205
Agarose MP	Sigma-Aldrich	Cat#11388983001
1 kb ladder	Agilent	Cat#201115
2.5 kb ladder	Bio-Rad	Cat#1708205
FACSVantage SE	BD Biosciences	https://www.bdbiosciences.com/en-us
FACSAria	BD Biosciences	https://www.bdbiosciences.com/en-us
Special Order Research Product FACSAria II	BD Biosciences	https://www.bdbiosciences.com/en-us
GC/MS (5873/6980)	Agilent	https://www.agilent.com
DB-17 column	Agilent	https://www.agilent.com
Tetrad2 Thermal Cycler	Bio-Rad	https://www.bio-rad.com
Typhoon FLA 9500 Phosphorimager	GE Healthcare	https://www.cytivalifesciences.com/

### Resource Availability

#### Lead Contact

Further information and requests for reagents and resources should be directed to and will be fulfilled by the Lead Contact, Kristin Ladell (ladellk@gmail.com).

#### Materials Availability

This study did not generate new unique reagents.

#### Data and Code Availability

The datasets reported in this study are available on request from the Lead Contact, Kristin Ladell (ladellk@gmail.com).

### Experimental Model and Subject Details

Three groups of human volunteers participated in this work. Cohort 1: volunteers with chronic HIV-1 infection (aged 36–53 years) were recruited for preliminary *in vivo* labeling studies (n = 4 males; Table S1). All were antiretroviral drug-free and seropositive for CMV. Cohort 2: healthy volunteers (aged 29–83 years) were recruited for more extensive *in vivo* labeling studies (n = 3 females; n = 5 males; Table S1). All were seronegative for hepatitis C virus and HIV-1 and seropositive for CMV. Cohort 3: additional healthy volunteers (aged 28–58 years) were recruited for phenotypic studies and measurements of telomere length and telomerase activity (n = 7 females; n = 7 males). Similar experiments were performed using venous blood samples donated by 5 of the 8 volunteers in cohort 2. All studies were conducted in accordance with the principles of the Declaration of Helsinki. Ethical approval was granted by the University of California Committee on Human Research (cohort 1), the London-Chelsea Research Ethics Committee (cohort 2), and the Cardiff University School of Medicine Research Ethics Committee (cohort 3).

### Method Details

#### Measurement and Analysis of Deuterium Enrichment in T Cell DNA

T cell proliferation *in vivo* was measured using deuterium (^2^H) labeling as described previously (Busch et al., 2007; Hellerstein et al., 1999; Ladell et al., 2008; McCune et al., 2000; Neese et al., 2001; Westera et al., 2013). Briefly, volunteers received heavy water (^2^H_2_O) orally for 7 weeks (Figure 1A), and deuterium incorporation into the DNA of flow-sorted T cells was quantified via gas chromatography/mass spectrometry (Agilent 5873/6980) (Ladell et al., 2008). DNA was released by boiling and hydrolyzed according to standard protocols, and deoxyribonucleosides were derivatized using pentafluorobenzyl hydroxylamine (Sigma-Aldrich). Gas chromatography/mass spectrometry was performed in negative chemical ionization mode using a DB-17 column (Agilent). The M+1/M+0 isotopomer ratio was monitored at mass-to-charge (*m/z*) 436/435. To normalize for body water enrichment, weekly saliva samples were analyzed for ^2^H_2_O content via calcium carbide-induced acetylene generation, monitoring at *m/z* 27/26 (Previs et al., 1996).

#### Flow Cytometry and Cell Sorting

T cell subsets of interest were flow-sorted from freshly isolated peripheral blood mononuclear cells (PBMCs) at >98% purity using a FACSVantage SE, a FACSAria, or a Special Order Research Product FACSAria II (all from BD Biosciences). Cells were stained with combinations of the following reagents: (1) anti-CD3–APC-H7 (clone SK7), anti-CD14–V500 (clone M5E2), anti-CD19–V500 (clone HIB19), anti-CD28–APC (clone CD28.2), anti-CD45RA–PE (clone HI100), anti-CD57–FITC (clone NK-1), anti-CCR7–FITC (clone 150503), and anti-CCR7–PE-Cy7 (clone 3D12) from BD Biosciences; (2) anti-CD4–PE-Cy5.5 (clone S3.5), anti-CD27–QD605 (clone CLB-27/1), and LIVE/DEAD Fixable Aqua from Thermo Fisher Scientific; (3) anti-CD8–BV711 (clone RPA-T8), anti-CD28–BV421 (clone CD28.2), anti-CD57–PE-Cy7 (clone NK-1), anti-CD127–BV421 (clone A019D5), anti-CCR7–BV421 (clone G043H7), anti-CXCR3–BV421 (clone G025H7), and anti-PD-1–BV421 (clone EH12.2H7) from BioLegend; and (4) anti-CD45RA–ECD (clone 2H4LDH11LDB9) and anti-CD45RO–ECD (clone UCHL1) from Beckman Coulter. Viable CD57^−^ and CD57^+^ memory T cells were identified in the CD4^+^ and/or CD8^+^ lineages after exclusion of CD27^bright^CD45RO^−^ (Figure 1C) or CD45RA^+^CCR7^+^ events (Figure S1). Cytosolic expression of Ki67 was evaluated using anti-Ki67–AF647 (clone B56; BD Biosciences) in conjunction with a Cytofix/Cytoperm Kit (BD Biosciences), and cytosolic/intranuclear expression of Ki67 was evaluated using anti-Ki67–FITC (clone B56; BD Biosciences) in conjunction with a Foxp3 Transcription Factor Staining Buffer Kit (Thermo Fisher Scientific). Data were analyzed with FlowJo software version 9.9.4 (FlowJo LLC).

#### Single Chromosome Telomere Length Analysis

DNA was extracted from 3,000 flow-sorted T cells using a QIAmp DNA Micro Kit (QIAGEN). Single telomere length analysis (STELA) was carried out at the XpYp or the 17p telomere as described previously (Capper et al., 2007). Briefly, 0.75 μL of the Telorette-2 linker (10 μM) was added to genomic DNA eluted in 35 μL of Tris (10 mM). Multiple PCRs were then performed for each test DNA. Each reaction was set up in a final volume of 10 μL containing 250 pg of DNA and the telomere-adjacent and Teltail primers at a final concentration of 0.5 μM in 75 mM Tris-HCl pH 8.8, 20 mM (NH_4_)_2_SO_4_, 0.01% Tween-20, and 1.5 mM MgCl_2_, with 0.5 U of a 10:1 mixture of Taq (Thermo Fisher Scientific) and Pwo polymerase (Sigma-Aldrich). The reactions were processed in a Tetrad2 Thermal Cycler (Bio-Rad). DNA fragments were resolved via 0.5% Tris-acetate-EDTA agarose gel electrophoresis and identified via Southern hybridization with a random-primed α-^33^P-labeled (PerkinElmer) TTAGGG repeat probe, together with probes specific for molecular weight markers at 1 kb (Agilent) and 2.5 kb (Bio-Rad). Hybridized fragments were detected using a Typhoon FLA 9500 Phosphorimager (GE Healthcare). The molecular weights of the DNA fragments were calculated using Phoretix 1D Quantifier (Nonlinear Dynamics).

#### Measurement of Telomerase Activity

Flow-sorted T cells were lyzed and assayed in two steps using a modified SYBR Green real-time quantitative telomeric repeat amplification protocol (Wege et al., 2003). Standard curves were obtained from serial dilutions of a 293T cell extract with known telomerase activity. Experimental telomerase activity was calculated with reference to 293T cells and expressed as relative telomerase activity (Ct_293T_/Ct_sample_).

### Quantification and Statistical Analysis

#### General Statistics

Unmatched groups were compared using the Mann-Whitney *U* test, and matched groups were compared using the paired samples Wilcoxon test. Significance was assigned at p < 0.05.

#### Mathematical Modeling

Mechanistic ordinary differential equation-based models were developed to assess the dynamics of CD57^−^ and CD57^+^ memory T cells (Costa Del Amo et al., 2018; Patel et al., 2017). These subsets were modeled as dependent populations to investigate the possibility that label acquisition in the CD57^+^ compartment was a consequence of proliferation-linked differentiation in the CD57^−^ compartment. Accordingly, CD57^−^ and CD57^+^ memory T cells were allowed to proliferate and die or exit the circulation, and CD57^−^ memory T cells were allowed to gain expression of CD57. The phenotypic conversion of CD57^−^ memory T cells into CD57^+^ memory T cells was considered over *n* rounds of division, including the possibility that *n* = 0.

In the applied model, CD57^−^ memory T cells (*x*_*1*_) became CD57^+^ memory T cells (*x*_*2*_) at a rate *m*:

$$\frac{dx_{1}}{dt}=p_{1}x_{1}-z_{1}x_{1}$$

$$\frac{dx_{2}}{dt}=p_{2}x_{2}-z_{2}x_{2}+mx_{1}$$

where *p*_*1*_ and *p*_*2*_ are the rates of proliferation of CD57^−^ and CD57^+^ memory T cells, respectively, and *z*_*1*_ and *z*_*2*_ are the rates of disappearance of CD57^−^ and CD57^+^ memory T cells, respectively. The possibility that surface expression of CD57 could be acquired during clonal expansion was accommodated in the permitted values for *m* (bounds during fitting [0,40]).

To minimize the number of free parameters, label enrichment among CD57^+^ memory T cells was assumed to originate either from dividing CD57^−^ memory T cells that differentiated into CD57^+^ memory T cells or from dividing CD57^+^ memory T cells. A model in which the acquisition of CD57 was not coincident with clonal expansion resulted in a substantially worse fit to the data and was not pursued further. The fraction of label thus became:

$$\frac{dL_{1}}{dt}=p_{1}b_{w}U_{t}-z_{1}^{*}L_{1}$$

$$\frac{dL_{2}}{dt}=\left(p_{2}+mR\right)b_{w}U_{t}-{\text{z}}_{2}^{*}L_{2}$$

where *p*_*1*_ and *p*_*2*_ are as above, *z*_*1*_*^∗^* and *z*_*2*_*^∗^* are the rates of loss of labeled CD57^−^ and CD57^+^ memory T cells, respectively, *L*_*1*_ and *L*_*2*_ are the fractions of labeled deoxyadenosine among CD57^−^ and CD57^+^ memory T cells, respectively, *R* is the ratio of CD57^−^ to CD57^+^ memory T cells (*x*_*1*_*/x*_*2*_), and *b*_*w*_ is the amplification factor estimated from label acquisition among granulocytes, assuming 100% turnover in 7 weeks. Data were available from 4 volunteers and gave a population average value for *b*_*w*_ of 3.5, consistent with previous studies (Ahmed et al., 2015; Lahoz-Beneytez et al., 2016). The value of *b*_*w*_ was therefore fixed at 3.5. Finally, *U(t)* is an empirical function used to describe the availability of label in body water:

$$U\left(t\right)=f\left(1-e^{-\delta t}\right)+\beta e^{-\delta t}\;\text{during}\;\text{labeling}\;t\leq \tau$$

$$U\left(t\right)=f\left(1-e^{-\delta t}\right)+\beta e^{-\delta t}\;\text{during}\;\text{labeling}\;t\leq \tau$$

as described previously (Vrisekoop et al., 2008), where *U(t)* represents the fraction of labeled precursor in body water at time *t* (in days), *f* is the fraction of labeled precursor in ingested water, *τ* is the length of the labeling period, δ is the turnover rate of body water per day, and *β* is the plasma enrichment attained at the end of day 0. Parameters were estimated by fitting the above functions to the deuterium labeling data measured in saliva. The resulting fits of *U(t)* to saliva measurements are shown in Figure S2.

#### Inclusion of Telomere Length Data in Model Fits

The impact of cell division on telomere length was modeled as described previously (De Boer and Noest, 1998). Telomere length data were available from 5 of the 8 volunteers in cohort 2. The model was fitted simultaneously to the labeling data and the telomere length data using the free parameters *p*_*1*_ and *p*_*2*_ to describe the rates of proliferation of CD57^−^ and CD57^+^ memory T cells, respectively, *z*_*1*_*^∗^* and *z*_*2*_*^∗^* to describe the rates of disappearance of labeled CD57^−^ and CD57^+^ memory T cells, respectively, and *mR*, the rate of conversion from CD57^−^ to CD57^+^ memory T cells (*m*) multiplied by the ratio of the frequency of CD57^−^ memory T cells to the frequency of CD57^+^ memory T cells (*R*).

#### Estimation of Telomere Length Change

The rate of change in telomere loss indices for CD57^−^ memory T cells was defined according to a previous report (De Boer and Noest, 1998) as follows:

$$\frac{d\mu_{1}}{dt}=2p_{1}$$

$$\frac{d\mu_{2}}{dt}=2p_{2}-\;mR\left(\mu_{2}-\mu_{1}-K\right)$$

where *p*_*1*_, *p*_*2*_, *m*, and *R* are as above, *K* is the length of telomere loss upon clonal expansion in units of division, and *μ*_*1*_ and *μ*_*2*_ are the average number of divisions undergone by CD57^−^ and CD57^+^ memory T cells, respectively. The difference in telomere length between CD57^−^ and CD57^+^ memory T cells was estimated as:

$$\Delta =\left(\mu_{2}-\mu_{1}\right)\varepsilon$$

where *ε* is the average number of base pairs (bp) lost per division (taken to be 50 bp [De Boer and Noest, 1998]), giving the following expression for the difference in telomere length:

$$\Delta_{C}=\varepsilon \left(\frac{2\left(p_{2}-p_{1}\right)}{mR}+K\right)\;$$

#### Fitting Procedure

The function *U(t)* was fitted to the deuterium labeling data measured in saliva, and the free parameters *f*, *β*, and *δ* were estimated for each individual. The resulting parameterized *U(t)* functions were then used as fixed inputs during simultaneous fitting of the deuterium labeling and telomere length data from CD57^−^ and CD57^+^ memory T cells using the equations for *L*_*1*_, *L*_*2*_, and *Δ*_*C*_ above. The free parameters were *p*_*1*_, *p*_*2*_, *z*_*1*_, *z*_*2*_, and *mR*. As telomerase is highly active during clonal expansion, the telomere length loss index (*K*) was initially set to 0 (Bodnar et al., 1996; Collins, 2006). This assumption was subsequently relaxed to explore the impact of variations in *K* (Figure S6). The contribution of self-renewal to the production of new CD57^+^ memory T cells was defined as:

$$\text{contribution}\;\text{from}\;\text{self-}\text{renewal}=\frac{p_{2}x_{2}}{p_{2}x_{2}+mx_{1}}=\frac{p_{2}}{p_{2}+mR}$$

To ensure that the labeling data and the telomere length data contributed equally to the fit, all residuals were normalized by the mean, and the deuterium residuals were divided by the number of labeling data points. Conclusions were analyzed for robustness against changes in the number of telomere base pairs lost per division. Scenarios in which CD57^+^ memory T cells did not proliferate were also tested by fixing *p*_*2*_ to 0. Model performance was evaluated using the F test and the AICc (Burnham and Anderson, 2002). The model was fitted to the data using non-linear least-squares regression implemented via the algorithm Pseudo in the FME package in R (Soetaert and Petzoldt, 2010).

## Supplemental Information

Document S1. Figures S1–S7 and Table S1

Document S2. Article plus Supplemental Information
