# Supplementary material for: CD57+ Memory T Cells Proliferate In Vivo
Source: Cell Rep. 2020 Dec 15;33(11):108501. doi: 10.1016/j.celrep.2020.108501 (PMC7758161; doi:10.1016/j.celrep.2020.108501)
Supplement: Document S1. Figures S1–S7 and Table S1 [file mmc1.pdf]

Cell Reports, Volume *33*

Supplemental Information

CD57$^+$ Memory T Cells Proliferate *In Vivo*

Raya Ahmed, Kelly L. Miners, Julio Lahoz-Beneytez, Rhiannon E. Jones, Laureline Roger, Christina Baboonian, Yan Zhang, Eddie C.Y. Wang, Marc K. Hellerstein, Joseph M. McCune, Duncan M. Baird, David A. Price, Derek C. Macallan, Becca Asquith, and Kristin Ladell

SUPPLEMENTAL MATERIAL

1. SUPPLEMENTAL FIGURES

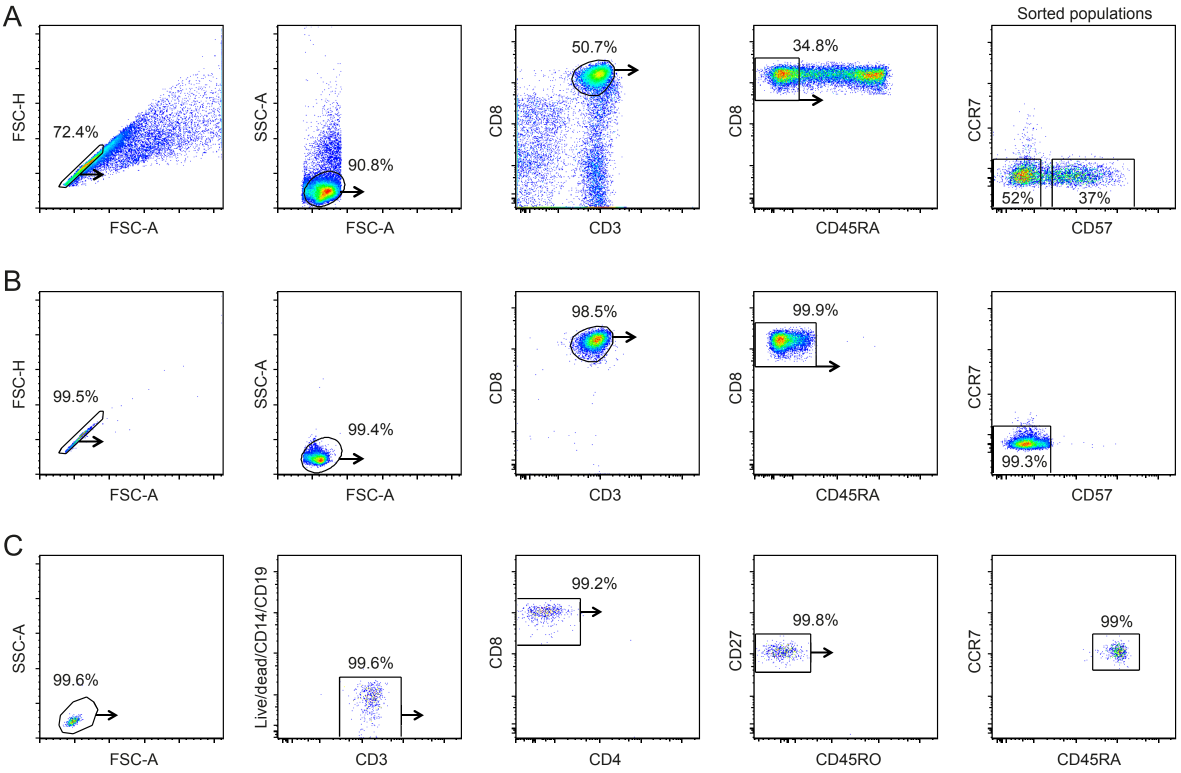

Figure S1. Flow cytometric gating strategy for the pilot ^2H_2O labeling study (Cohort 1) and representative sort purity checks (Cohorts 1 and 2). (A) Successive panels depict the flow cytometric gating strategy used to sort CD57$^-$ and CD57$^+$ memory CD8$^+$ T cells (Cohort 1). Single cells were identified in a forward scatter-area versus forward scatter-height plot, and lymphocytes were identified in a forward scatter-area versus side scatter-area plot. Sort gates were then fixed on CD3$^+$CD8$^+$CD45RA$^-$CCR7$^-$CD57$^-$ and CD3$^+$CD8$^+$CD45RA$^-$CCR7$^-$CD57$^+$ memory T cells. (B) Representative sort purity check for CD3$^+$CD8$^+$CD45RA$^-$CCR7$^-$CD57$^-$ memory T cells (Cohort 1). (C) Representative sort purity check for CD3$^+$CD8$^+$CD27$^+$CD45RO$^-$CCR7$^+$ naive T cells (Cohort 2). This abundant subset was analyzed to preserve CD57$^-$ and CD57$^+$ memory T cells. Related to Figure 1.

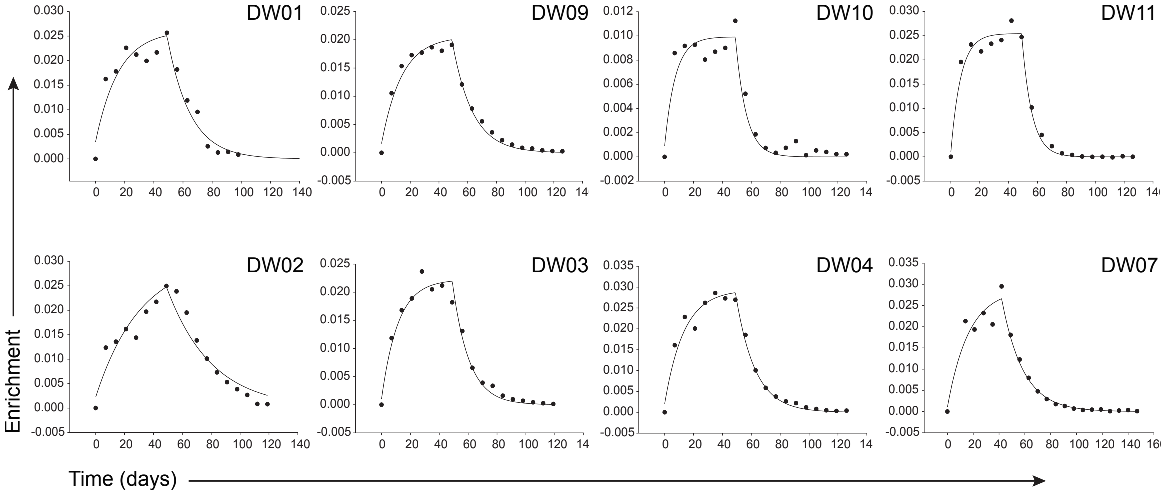

Figure S2. ^{2}H enrichment in body water. Measured ^{2}H enrichments in body water (dots) with best-fit curves (solid lines) for all volunteers in Cohort 2. Young volunteers are shown on the top row, and elderly volunteers are shown on the bottom row. Related to Figure 1.

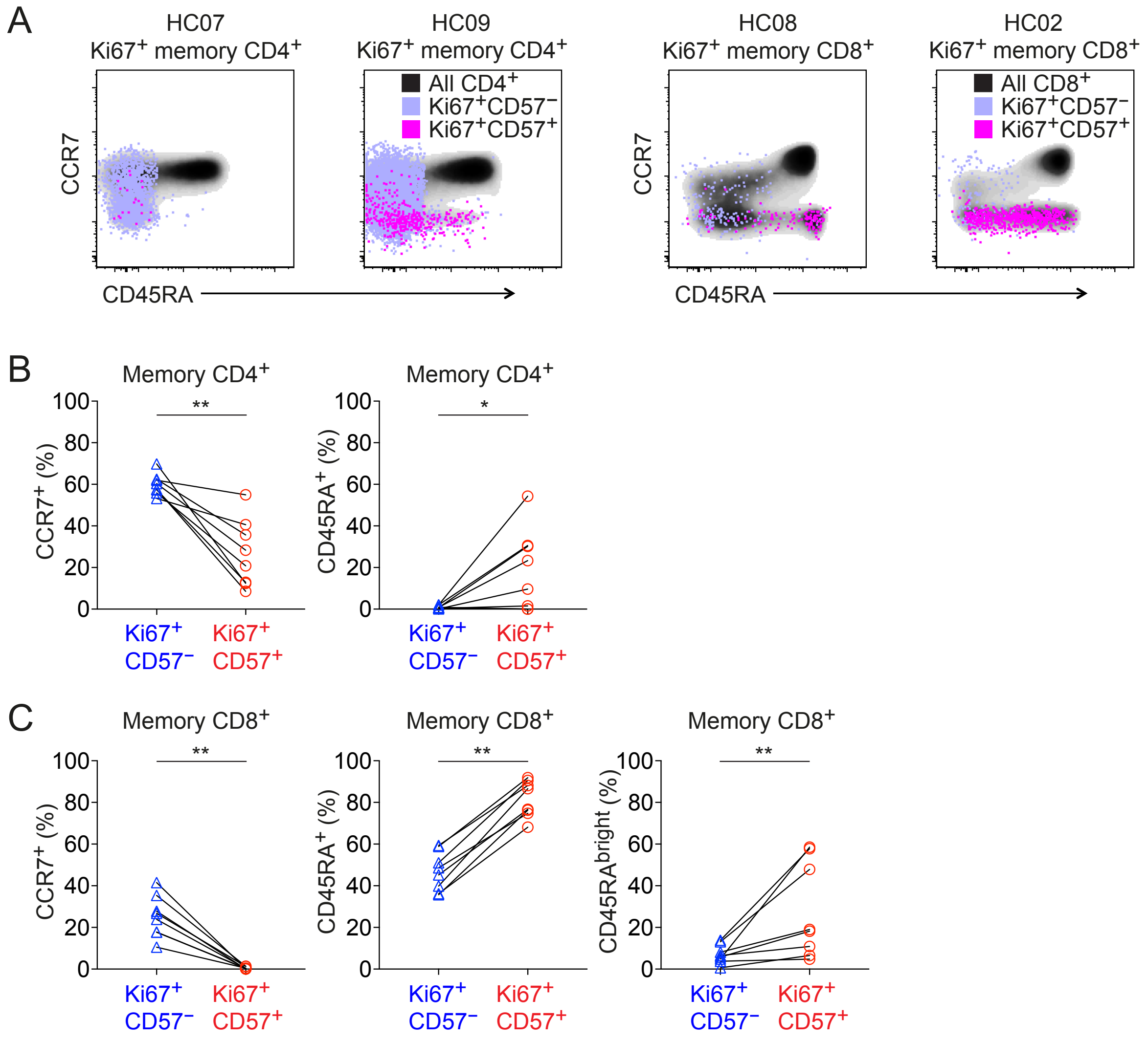

Figure S3. Expression of Ki67 among $CD57^-$ and $CD57^+$ memory T cells. (A) Representative flow cytometric data from unlabeled volunteers (n = 4) showing the phenotypic characteristics of $Ki67^+CD57^-$ and $Ki67^+CD57^+$ memory $CD4^+$ (left panels) or $CD8^+$ T cells (right panels) overlaid on density clouds representing the corresponding total $CD4^+$ (left panels) or $CD8^+$ T cell populations (right panels). HC02 was seropositive for CMV. HC07 and HC08 were seronegative for CMV. (B) Percent cytosolic/nuclear expression of CCR7 (left) and CD45RA (right) among $Ki67^+CD57^-$ (blue triangles) and $Ki67^+CD57^+$ memory $CD4^+$ T cells (red circles). (C) Percent cytosolic/nuclear expression of CCR7 (left) and CD45RA (center and right) among $Ki67^+CD57^-$ (blue triangles) and $Ki67^+CD57^+$ memory $CD8^+$ T cells (red circles). The right graph shows only $CD45RA^{bright}$ events. *$p < 0.05$, **$p < 0.01$. Paired samples Wilcoxon test. Related to Figure 2.

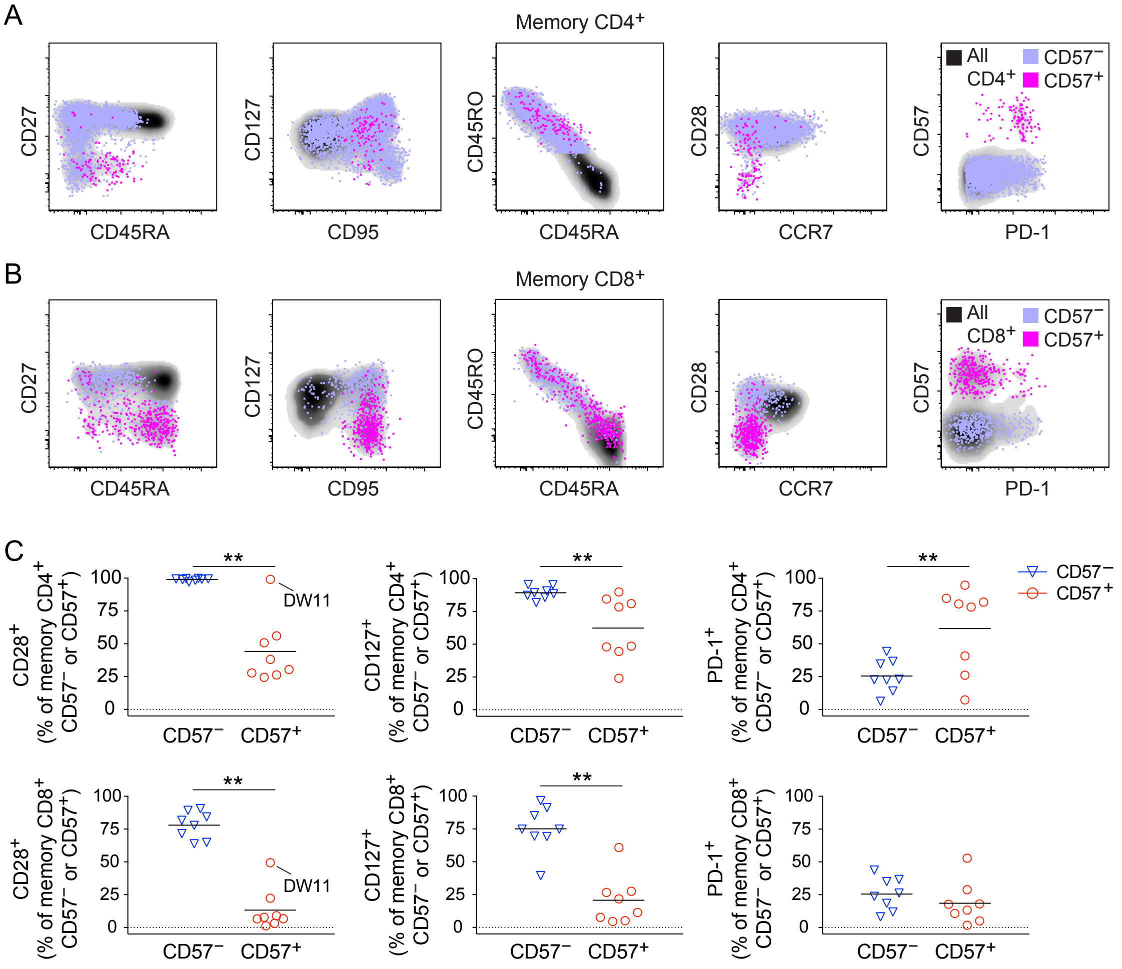

Figure S4. **CD57$^+$ memory T cells are phenotypically distinct in the CD4$^+$ and CD8$^+$ lineages.** (A) Phenotypic characteristics of CD57$^-$ and CD57$^+$ memory CD4$^+$ T cells from a labeled volunteer (DW01) shown overlaid on density clouds representing the corresponding total CD4$^+$ T cell population. (B) Phenotypic characteristics of CD57$^-$ and CD57$^+$ memory CD8$^+$ T cells from a labeled volunteer (DW01) shown overlaid on density clouds representing the corresponding total CD8$^+$ T cell population. (C) Percent expression of CD28 (left), CD127 (center), and PD-1 (right) among CD57$^-$ and CD57$^+$ memory CD4$^+$ (top) or CD8$^+$ T cells (bottom) sampled from all volunteers in Cohort 2. Unusually high frequencies of CD57$^+$ memory CD4$^+$ and CD57$^+$ memory CD8$^+$ T cells expressed CD28 in one volunteer (DW11). **$p < 0.01$. Paired samples Wilcoxon test. Related to Figure 2.

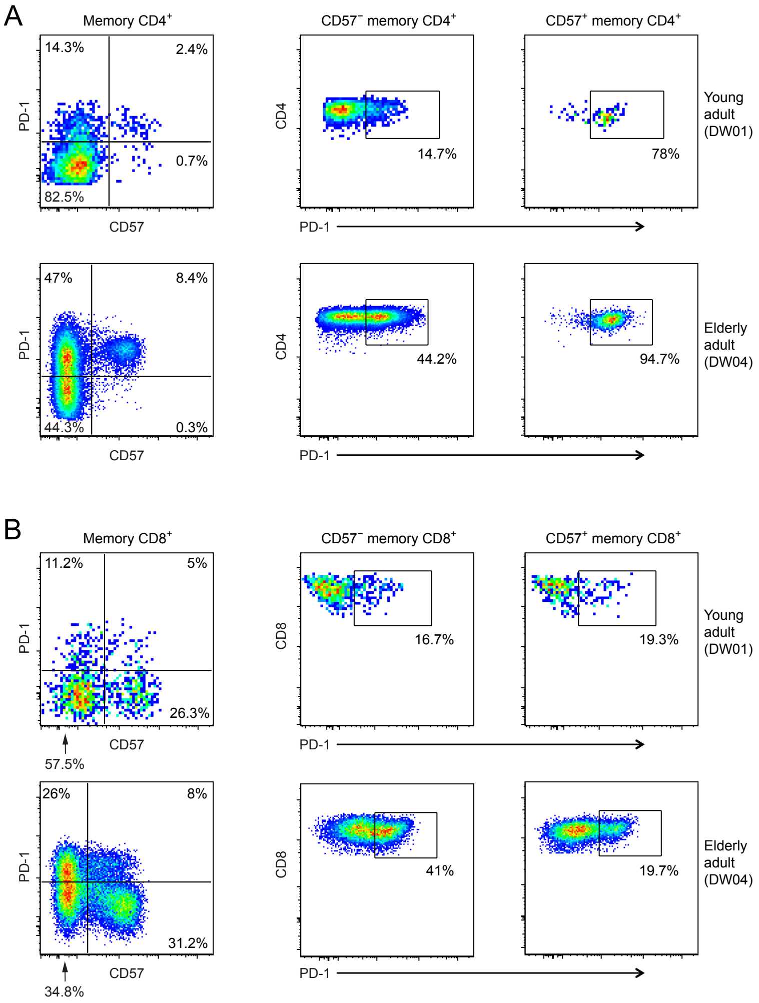

Figure S5. Expression of PD-1 among CD57$^-$ and CD57$^+$ memory T cells. (A) Representative flow cytometric data showing the expression of PD-1 among CD57$^-$ and CD57$^+$ memory CD4$^+$ T cells. A young volunteer is shown on the top row (DW01). An elderly volunteer is shown on the bottom row (DW04). (B) Representative flow cytometric data showing the expression of PD-1 among CD57$^-$ and CD57$^+$ memory CD8$^+$ T cells. A young volunteer is shown on the top row (DW01). An elderly volunteer is shown on the bottom row (DW04). Related to Figure 2.

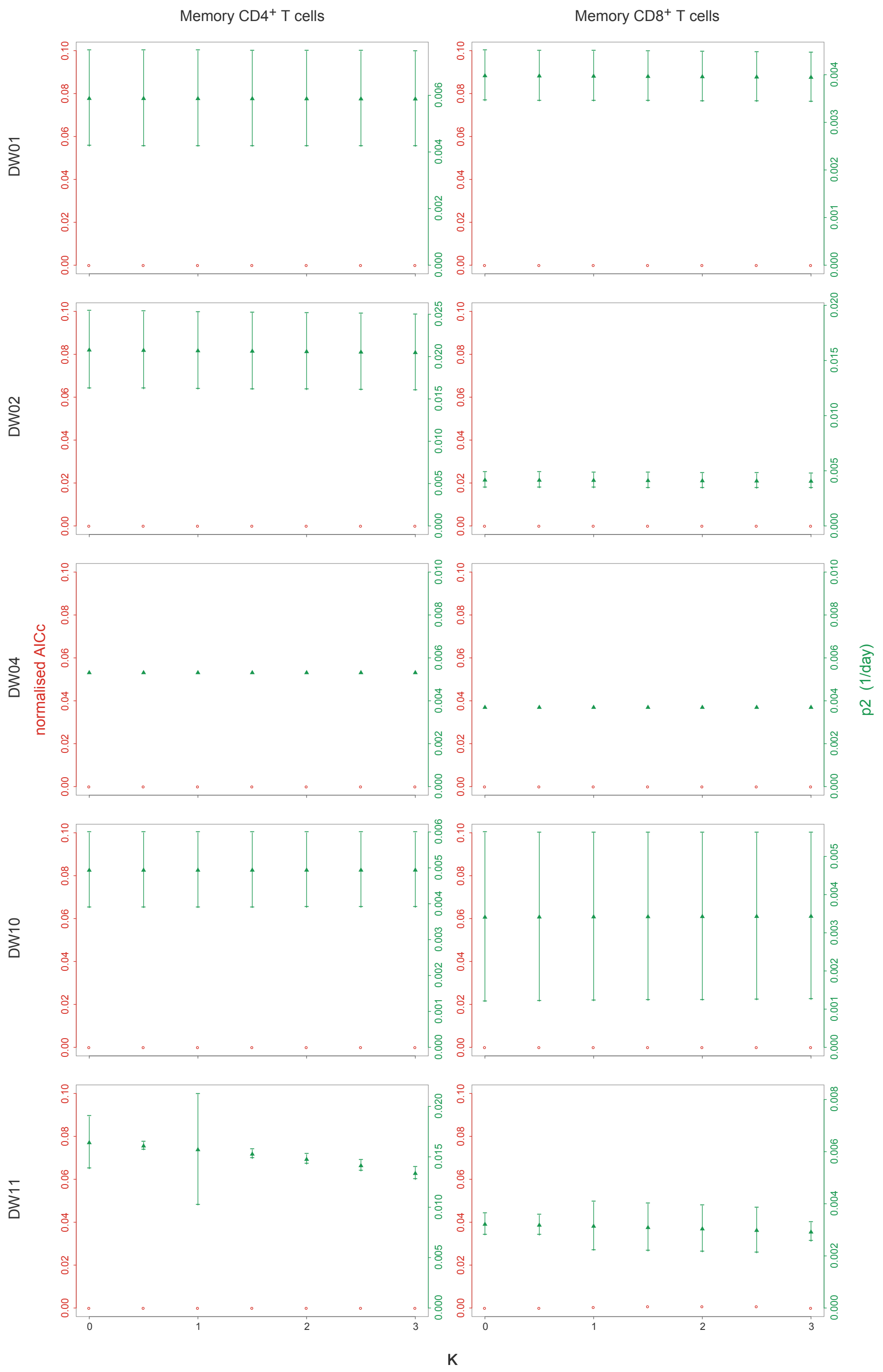

Figure S6. Sensitivity of parameter estimates and AICc values to changes in the telomere loss index shift (*K*). The telomere loss index shift, defined as the length of telomere lost per unit of division, is shown on the x-axes (denoted as *K*). Each plot shows the impact of varying *K* on the normalized AICc (red circles) and the estimated rate of proliferation in the corresponding CD57$^+$ memory T cell population (green triangles). Error bars show 95% confidence intervals. Normalized AICc = AICc − AICc of the winning model. Related to Figure 4.

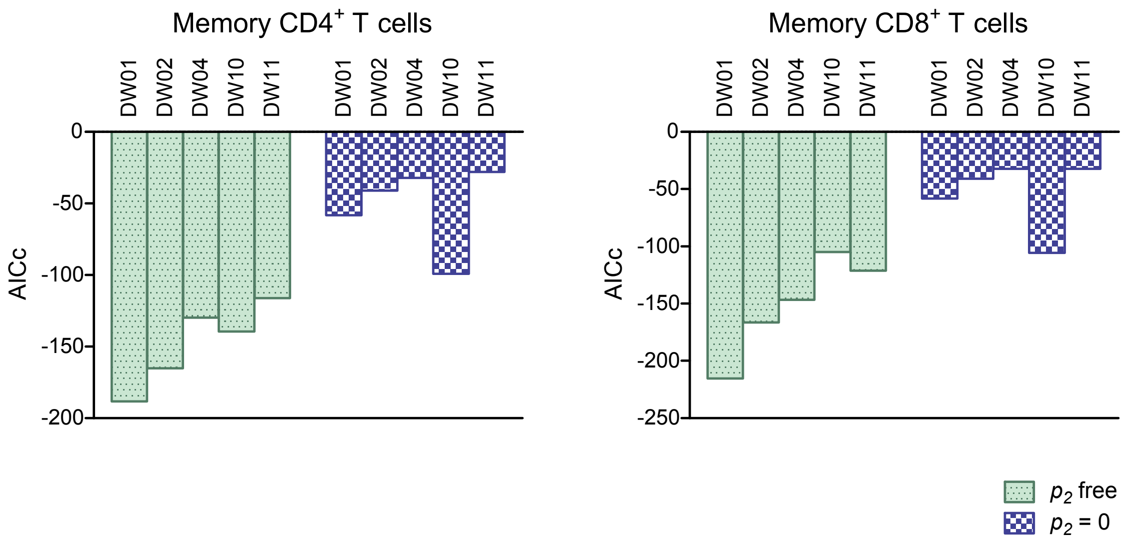

Figure S7. Quality of model fits to the experimental data with p_2 free or constrained to zero. Quality of fit was assessed using the AICc. Lower values indicate a better quality of fit, and higher values indicate a worse quality of fit. The proliferation rate in each $CD57^+$ memory T cell population (p_2) was either free (green) or constrained to zero (blue). Related to Figure 4.

2. SUPPLEMENTAL TABLES

ID	Age	Gender	HIV-1 load (copies/mL plasma)	$CD4^+$ T cell count ($x10^9/L$)	$CD8^+$ T cell count ($x10^9/L$)	CMV serology	HIV-1 serology
HI16	53	Male	74,199	0.330	1.397	+	+
HI17	40	Male	2,059	0.852	3.388	+	+
HI19	36	Male	448,343	0.349	2.445	+	+
HI20	38	Male	30,851	0.431	1.646	+	+
DW01	32	Female	NA	0.809	0.460	+	−
DW02	64	Male	NA	0.681	0.314	+	−
DW03	78	Male	NA	0.869	0.405	+	−
DW04	83	Male	NA	0.885	0.579	+	−
DW07	60	Female	NA	0.654	0.241	+	−
DW09	47	Male	NA	1.077	0.800	+	−
DW10	34	Male	NA	0.488	0.977	+	−
DW11	29	Female	NA	1.380	0.864	+	−

Table S1. Clinical details and demographics of HIV-1-infected (HI) and healthy volunteers (DW) included in the labeling studies. NA, not applicable. Related to Figures 1–4.
