# Supplementary material for: CD57+ Memory T Cells Proliferate In Vivo
Source: Cell Rep. 2020 Dec 15;33(11):108501. doi: 10.1016/j.celrep.2020.108501 (PMC7758161; doi:10.1016/j.celrep.2020.108501)
Supplement: Document S2. Article plus Supplemental Information [file mmc2.pdf]

Cell Reports

Report

CD57+ Memory T Cells Proliferate *In Vivo*

Graphical Abstract

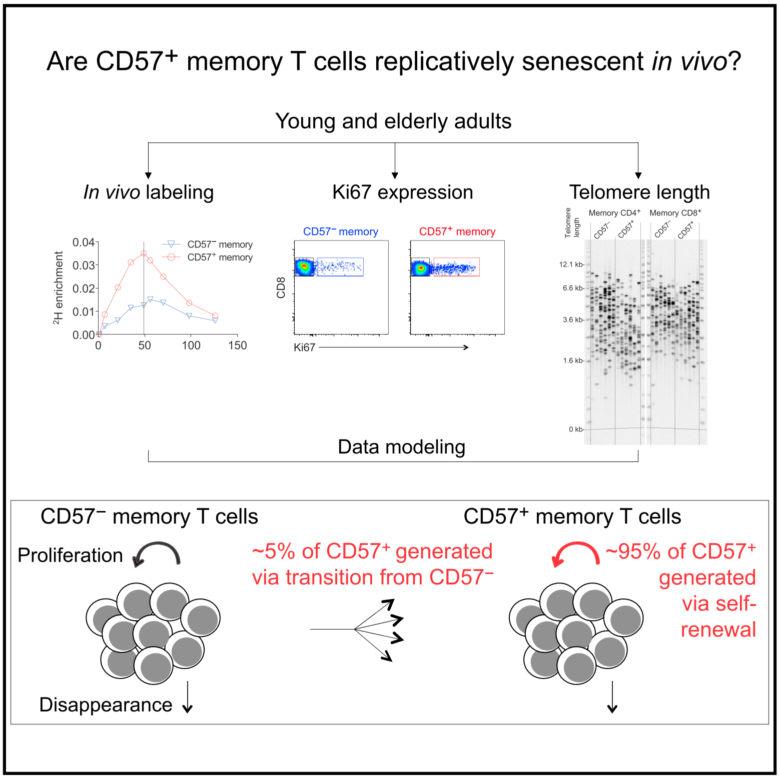

Highlights

- CD57+ memory T cells are not replicatively senescent *in vivo*
- CD57+ memory T cells are maintained primarily via self-renewal *in vivo*

Authors

Raya Ahmed, Kelly L. Miners, Julio Lahoz-Beneytez, ..., Derek C. Macallan, Becca Asquith, Kristin Ladell

Correspondence

priced6@cardiff.ac.uk (D.A.P.), macallan@sgul.ac.uk (D.C.M.), b.asquith@imperial.ac.uk (B.A.), ladellk@gmail.com (K.L.)

In Brief

In vitro studies have led to the widely held assumption that replicatively senescent memory T cells express the carbohydrate epitope CD57. Using a variety of experimental approaches and mathematical modeling of composite datasets, Ahmed et al. show that CD57+ memory T cells proliferate and self-renew *in vivo*.

Ahmed et al., 2020, Cell Reports *33*, 108501
December 15, 2020 © 2020 The Authors.
https://doi.org/10.1016/j.celrep.2020.108501

CellPress

Cell Reports

CellPress
OPEN ACCESS

Report

CD57^{+} Memory T Cells Proliferate *In Vivo*

Raya Ahmed,[1,10] Kelly L. Miners,[2,10] Julio Lahoz-Beneytez,[3,10] Rhiannon E. Jones,[4] Laureline Roger,[2] Christina Baboonian,[1] Yan Zhang,[1] Eddie C.Y. Wang,[2] Marc K. Hellerstein,[5] Joseph M. McCune,[6] Duncan M. Baird,[4] David A. Price,[2,7,10,*] Derek C. Macallan,[1,8,10,*] Becca Asquith,[3,10,*] and Kristin Ladell[2,9,10,11,*]
[1]Institute for Infection and Immunity, St. George's, University of London, London SW17 0RE, UK
[2]Division of Infection and Immunity, Cardiff University School of Medicine, Heath Park, Cardiff CF14 4XN, UK
[3]Department of Infectious Disease, Imperial College London, London W2 1PG, UK
[4]Division of Cancer and Genetics, Cardiff University School of Medicine, Heath Park, Cardiff CF14 4XN, UK
[5]Department of Nutritional Sciences and Toxicology, University of California, Berkeley, CA 94720, USA
[6]HIV Frontiers Program, Global Health Innovative Technology Solutions, Bill & Melinda Gates Foundation, Seattle, WA 98109, USA
[7]Systems Immunity Research Institute, Cardiff University School of Medicine, Heath Park, Cardiff CF14 4XN, UK
[8]St George's University Hospitals NHS Foundation Trust, London SW17 0QT, UK
[9]Neonatal Unit, Singleton Hospital, Swansea Bay University Health Board, Swansea SA2 8QA, UK
[10]These authors contributed equally
[11]Lead Contact
*Correspondence: priced6@cardiff.ac.uk (D.A.P.), macallan@sgul.ac.uk (D.C.M.), b.asquith@imperial.ac.uk (B.A.), ladellk@gmail.com (K.L.)
https://doi.org/10.1016/j.celrep.2020.108501

SUMMARY

A central paradigm in the field of lymphocyte biology asserts that replicatively senescent memory T cells express the carbohydrate epitope CD57. These cells nonetheless accumulate with age and expand numerically in response to persistent antigenic stimulation. Here, we use *in vivo* deuterium labeling and *ex vivo* analyses of telomere length, telomerase activity, and intracellular expression of the cell-cycle marker Ki67 to distinguish between two non-exclusive scenarios: (1) CD57^{+} memory T cells do not proliferate and instead arise via phenotypic transition from the CD57^{-} memory T cell pool; and/or (2) CD57^{+} memory T cells self-renew via intracompartmental proliferation. Our results provide compelling evidence in favor of the latter scenario and further suggest in conjunction with mathematical modeling that self-renewal is by far the most abundant source of newly generated CD57^{+} memory T cells. Immunological memory therefore appears to be intrinsically sustainable among highly differentiated subsets of T cells that express CD57.

INTRODUCTION

Immune senescence has been linked with the accumulation of terminally differentiated lymphocytes that fail to proliferate in response to antigenic challenge. It has also been suggested that surface expression of CD57, a terminally sulfated glycan carbohydrate epitope (Abo and Balch, 1981), identifies memory T cells that lack the capacity to proliferate (Brenchley et al., 2003). In line with these widely accepted paradigms, highly differentiated effector memory T cells that express CD45RA, known as T_{EMRA} cells, become more prevalent with age (Nociari et al., 1999) and often express CD57 (Ladell et al., 2008).

Contrary to the notion of differentiation-linked senescence, memory T cells that express CD57 can be induced to proliferate *in vitro*, at least under optimized conditions (Chong et al., 2008; Izquierdo et al., 1990). Parallel strands of evidence have further suggested a key role for these cells as immune effectors. For example, memory CD8^{+} T cells rarely express CD57 in conjunction with programmed death-1 (PD-1) (Petrovas et al., 2009), a marker associated with exhaustion (Day et al., 2006; Freeman et al., 2006; Petrovas et al., 2006; Trautmann et al., 2006), and functionally replete memory CD4^{+} and CD8^{+} T cells with cytotoxic potential typically express high levels of CD57 (Casazza et al., 2006; Chattopadhyay et al., 2009; Chong et al., 2008; Kern et al., 1999; Le Priol et al., 2006; Takata and Takiguchi, 2006; van Leeuwen et al., 2002). Virus-specific memory T cells have also been identified in the T_{EMRA} compartment (Appay et al., 2008). In the CD8^{+} lineage, these cells have been associated with protective effects, most notably during acute (Northfield et al., 2007) and chronic human immunodeficiency virus type 1 (HIV-1) infection (Addo et al., 2007), and in a study of elite controllers with eventual disease progression, increasing levels of viral replication appeared to drive the formation of CD28^{-}CD57^{+} memory CD8^{+} T cells, potentially indicating a reactive escalation in the cytotoxic response to HIV-1 (Benito et al., 2018). Accordingly, CD57^{+} memory T cells enrich the immune system with important antigen-dependent effector functions and, by extension, do not necessarily represent an irrelevant "cul-de-sac" in the lymphocyte differentiation pathway.

In this study, we used deuterium labeling to quantify the proliferation of CD57^{-} and CD57^{+} memory T cells *in vivo* and supplemented these analyses with *ex vivo* measurements of telomere length, telomerase activity, and intracellular expression of the cell-cycle marker Ki67. We then used mathematical modeling to evaluate two non-exclusive hypothetical scenarios: (1) CD57^{+} memory T cells arise from the CD57^{-} memory T cell

Check for updates

This is an open access article under the CC BY license (http://creativecommons.org/licenses/by/4.0/).

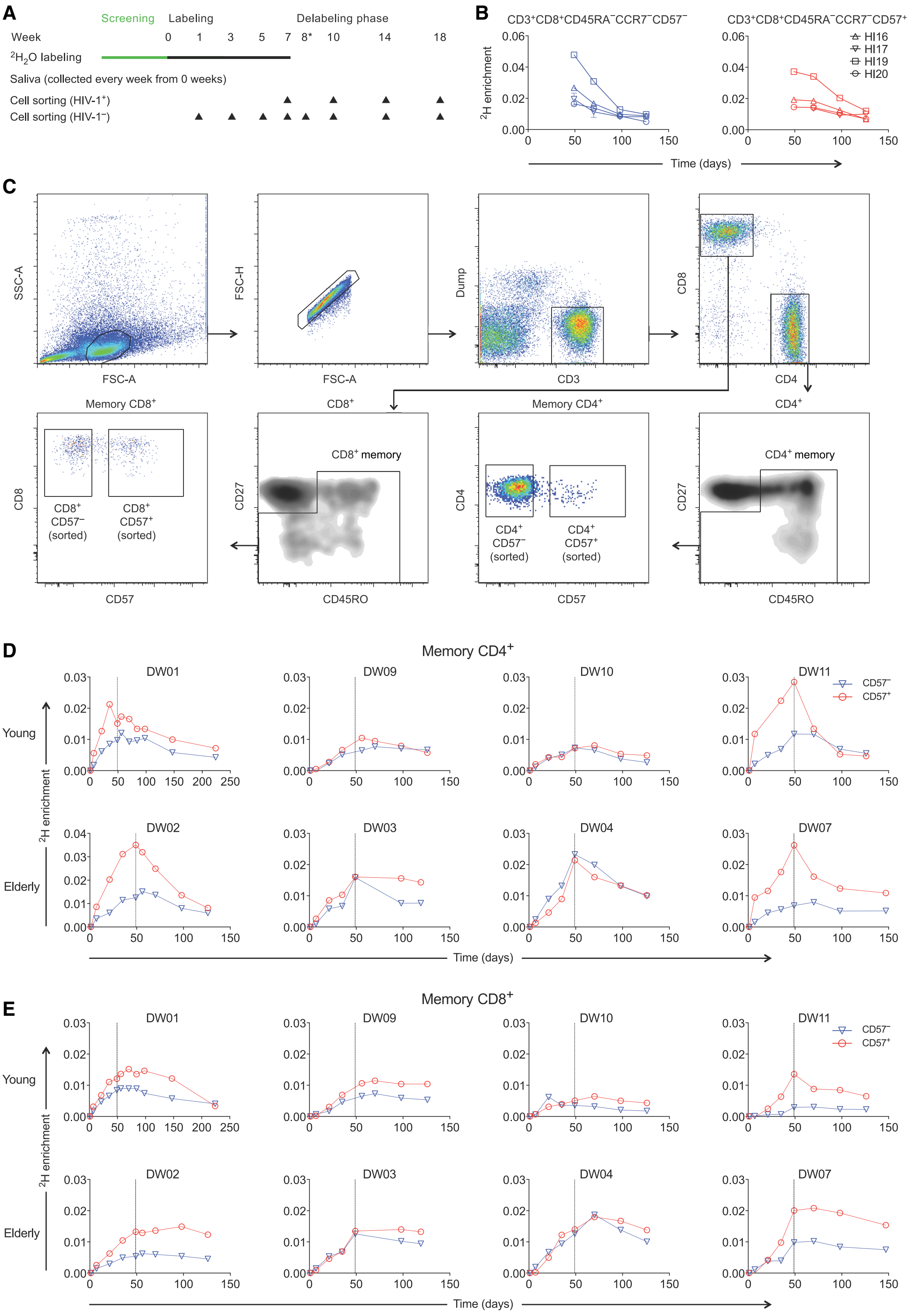

(legend on next page)

compartment as a consequence of progressive differentiation; and/or (2) $CD57^+$ memory T cells self-renew via intracompartmental proliferation and thereby contribute to long-term immunological memory.

RESULTS

$CD57^-$ and $CD57^+$ Memory T Cells Exhibit Similar Rates of Deuterium Incorporation

Preliminary *in vivo* labeling data were derived from studies of volunteers with chronic HIV-1 infection (aged 36–53 years), all of whom were antiretroviral drug-free at the time of experimentation and seropositive for cytomegalovirus (CMV; n = 4; Table S1). The labeling protocol is outlined in Figure 1A. Venous blood was sampled at weeks 7 (end of labeling), 10, 14, and 18, and at each time point, $CD57^-$ and $CD57^+$ memory $CD8^+$ T cells were flow-sorted from the $CD45RA^-CCR7^-$ subset at >98% purity (Figure S1). This gating strategy was designed to exclude T_{EMRA} cells, which were assessed separately in an earlier report (Ladell et al., 2008). Considerable rates of ^2H labeling and delabeling were observed among $CD45RA^-CCR7^-CD57^-$ and $CD45RA^-CCR7^-CD57^+$ memory $CD8^+$ T cells (Figure 1B).

Immune activation enhances the turnover of memory T cells in the setting of chronic HIV-1 or HIV-2 infection (Hegedus et al., 2014; McCune et al., 2000; Vrisekoop et al., 2015; Zhang et al., 2013). We therefore sought to confirm these preliminary findings in a more comprehensive labeling study of healthy volunteers (aged 29–83 years), all of whom were seronegative for HIV-1 and seropositive for CMV. Recruitment was stratified to include equal numbers of young (aged 29–47 years) and elderly individuals (aged 60–83 years), the latter representing a population in which immune senescence was more likely (total n = 8; Table S1). Venous blood was sampled during the labeling phase (weeks 1, 3, and 5), at the end of labeling (week 7), and during the delabeling phase (weeks 8, 10, 14, and 18) (Figure 1A). At each time point, $CD57^-$ and $CD57^+$ memory T cells were flow-sorted from the $CD4^+$ and $CD8^+$ lineages at >98% purity after gating out potentially naive $CD27^{bright}CD45RO^-$ events (Figures 1C and S1).

In each coreceptor-defined lineage, similar patterns of ^2H labeling and delabeling were observed among $CD57^-$ and $CD57^+$ memory T cells, and equivalent (n = 3) or greater rates of ^2H labeling (n = 5) were observed among $CD57^+$ memory T cells compared with $CD57^-$ memory T cells (Figures 1D and 1E). Importantly, the corresponding ^2H label enrichments in body water followed an expected rise-and-fall profile (Figure S2), and in the context of age-related immune senescence, no intralineage or intrasubset differences in the kinetics of ^2H accumulation or loss were apparent between young and elderly volunteers (Figures 1D and 1E).

$Ki67^+$ Cells Are Readily Detectable in the $CD57^+$ Memory T Cell Pool

To corroborate these findings, we measured the expression of Ki67, an intracellular marker that accumulates during active phases of the cell cycle (Gerdes et al., 1983; Miller et al., 2018). Cytosolic expression of Ki67 was detected in the $CD4^+$ lineage at mean frequencies of 1% among $CD57^-$ memory T cells and 2.9% among $CD57^+$ memory T cells (p = 0.02, paired samples Wilcoxon test; Figures 2A and 2B) and in the $CD8^+$ lineage at mean frequencies of 0.7% among $CD57^-$ memory T cells and 0.4% among $CD57^+$ memory T cells (p = 0.008, paired samples Wilcoxon test; Figures 2A and 2B). Higher frequencies were observed using a different approach that simultaneously exposed intranuclear antigens. Cytosolic/nuclear expression of Ki67 was detected in the $CD4^+$ lineage at mean frequencies of 4.9% among $CD57^-$ memory T cells and 8.6% among $CD57^+$ memory T cells (p = 0.742, paired samples Wilcoxon test; Figures 2C and 2D) and in the $CD8^+$ lineage at mean frequencies of 1.2% among $CD57^-$ memory T cells and 1.9% among $CD57^+$ memory T cells (p = 0.039, paired samples Wilcoxon test; Figures 2E and 2F).

In further analyses, we assessed the phenotypic characteristics of $Ki67^+CD57^-$ and $Ki67^+CD57^+$ memory T cells in the $CD4^+$ and $CD8^+$ lineages. As expected, $Ki67^+$ memory $CD4^+$ T cells predominantly expressed CD45RO, with or without CD57, whereas $Ki67^+$ memory $CD8^+$ T cells were phenotypically more heterogeneous and often expressed CD45RA in conjunction with CD57 (Figure 2G). Moreover, $Ki67^+CD57^-$ memory T cells expressed CD28 at higher frequencies than $Ki67^+CD57^+$ memory T cells, both in the $CD4^+$ lineage (p = 0.008, paired samples Wilcoxon test; Figures 2D and 2H) and in the $CD8^+$ lineage (p = 0.008, paired samples Wilcoxon test; Figures 2F and 2I). Similar patterns of expression were observed for CCR7 (Figure S3).

To link these findings with the labeling data, we compared the phenotypic characteristics of $Ki67^+CD57^-$ and $Ki67^+CD57^+$ memory T cells with the phenotypic characteristics of $CD57^-$ and $CD57^+$ memory T cells sampled from the healthy volunteers in cohort 2. In both coreceptor-defined lineages, $CD57^-$ memory T cells expressed CD28 and CCR7 at higher frequencies than $CD57^+$ memory T cells, akin to the corresponding $Ki67^+$ memory T cells (Figure S4). Of note, $CD57^+$ memory $CD4^+$ T cells mostly lacked CD27 but commonly expressed CD127 and PD-1, whereas $CD57^+$ memory $CD8^+$ T cells were generally more

Figure 1. $CD57^-$ and $CD57^+$ Memory T Cells Exhibit Similar Rates of Deuterium Incorporation

(A) Schematic representation of the ^2H_2O labeling protocol and sampling time points.

(B) Experimental labeling data for $CD57^-$ and $CD57^+$ memory $CD8^+$ T cells sampled from the HIV-1-infected volunteers in cohort 1. The corresponding flow cytometric gating strategy is shown in Figure S1.

(C) Successive panels depict the flow cytometric gating strategy used to sort $CD57^-$ and $CD57^+$ memory T cells from the $CD4^+$ and $CD8^+$ lineages (cohort 2). Lymphocytes were identified in a forward scatter-area versus side scatter-area plot, and single cells were identified in a forward scatter-area versus forward scatter-height plot. Boolean gates were drawn for analysis only to exclude fluorochrome aggregates. Viable $CD3^+CD14^-CD19^-$ cells were then identified in the $CD4^+$ and $CD8^+$ lineages, and sort gates were fixed on $CD57^-$ and $CD57^+$ memory cells after exclusion of potentially naive $CD27^{bright}CD45RO^-$ cells.

(D) Experimental labeling data for $CD57^-$ and $CD57^+$ memory $CD4^+$ T cells sampled from the healthy volunteers in cohort 2.

(E) Experimental labeling data for $CD57^-$ and $CD57^+$ memory $CD8^+$ T cells sampled from the healthy volunteers in cohort 2.

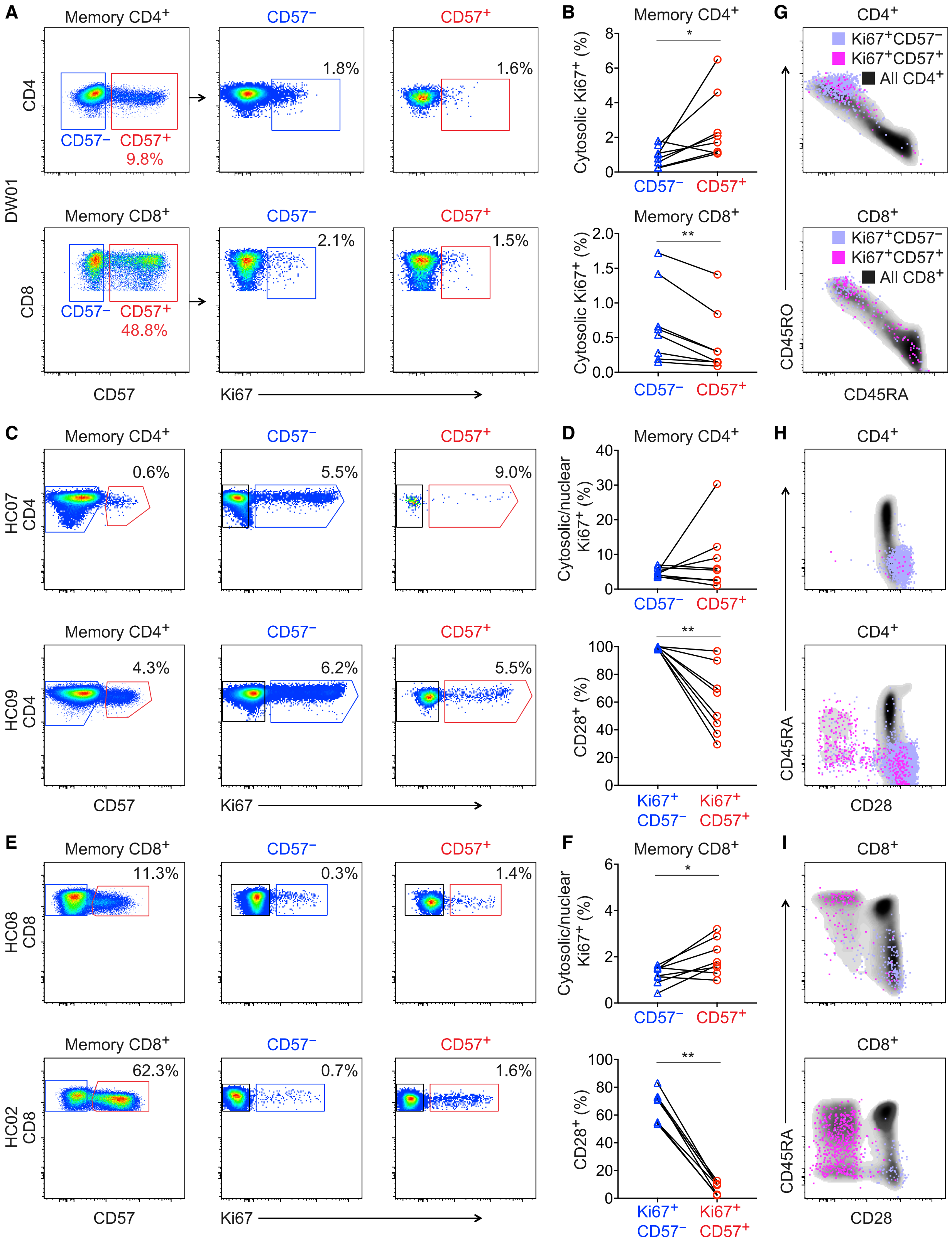

(legend on next page)

CellPress
OPEN ACCESS

differentiated and rarely expressed CD27, CD127, or PD-1 (Figures S4 and S5). Age had no apparent influence on these phenotypic characteristics (data not shown).

CD57$^-$ and CD57$^+$ Memory T Cells Have Similar Division Histories

To refine our understanding of these datasets, we measured XpYp or 17p telomere lengths in the CD57$^-$ and CD57$^+$ memory T cell pools (Figure 3A). Telomere lengths were distributed in a heterogeneous manner and overlapped considerably across CD57-defined subsets in the CD4$^+$ and CD8$^+$ lineages. In some volunteers, significant differences in mean telomere length were observed between the CD57$^-$ and CD57$^+$ memory T cell populations, most commonly in the CD4$^+$ lineage, but no consistent directional change was apparent between CD57-defined subsets in either the CD4$^+$ or the CD8$^+$ lineage (Figures 3B and 3C). However, pooling the XpYp data from labeled volunteers revealed that telomere lengths were maintained to a slightly greater extent in the CD57$^-$ memory CD4$^+$ T cell population compared with the CD57$^+$ memory CD4$^+$ T cell population (Figure 3D), and pooling the 17p data from volunteers in cohort 3 yielded a similar result with borderline significance ($p = 0.037$, Mann-Whitney *U* test; data not shown). Telomerase activity was generally low, as expected given the infrequent expression of Ki67, but marginally higher levels were detected among CD57$^-$ memory T cells compared with CD57$^+$ memory T cells in both coreceptor-defined lineages. These data were reported previously for reference in another labeling study of the volunteers in cohort 2 (Ahmed et al., 2016).

CD57$^-$ and CD57$^+$ Memory T Cells Self-Renew *In Vivo*

To integrate these findings, we fitted mathematical models simultaneously to the ^{2}H enrichment data and the telomere length data, allowing CD57$^-$ memory T cells to become CD57$^+$ memory T cells in the CD4$^+$ and CD8$^+$ lineages (Figure 4A). This approach was designed to capture both possible explanations for the accumulation of label in the corresponding CD57$^+$ compartments, namely that CD57$^-$ memory T cells proliferated and acquired expression of CD57 and/or that CD57$^+$ memory T cells proliferated and retained expression of CD57. Proliferation rates were denoted by p_1 and p_2 for CD57$^-$ and CD57$^+$ memory T cells, respectively, such that replicative senescence in the CD57$^+$ subsets was represented by the constraint $p_2 = 0$. Telomeres shorten by an average of 50 bp per cell division (De Boer and Noest, 1998). In some cases, this rate of erosion can be counteracted by the activity of telomerase, a possibility that was included in the model assumptions via an additional parameter, termed K.

Fits were restricted to the volunteers for whom labeling data and telomere length data were available ($n = 5$). The general model (with p_2 free) fitted the data well for CD57$^-$ and CD57$^+$ memory T cells in the CD4$^+$ and CD8$^+$ lineages (Figure 4B; Table 1). Importantly, the proliferation rate estimates for the CD57$^+$ subsets were positive, with 95% confidence intervals that did not overlap zero (i.e., $p_2 > 0$). This conclusion was robust to different rates of telomere shortening per cell division and changes in the parameter K (Figure S6). Model performance was considerably worse if proliferation was disallowed in the CD57$^+$ memory T cell populations (i.e., $p_2 = 0$) (Figures 4B and S7). Indeed, the median p value in a comparison of the models was 5×10^{-20} (F test), which provided strong evidence to reject the null hypothesis of the simpler model, namely that CD57$^+$ memory T cells were unable to proliferate (i.e., $p_2 = 0$). Moreover, the median small-sample-corrected Akaike information criterion (AICc) difference was 106, which indicated that the simpler model provided a substantially worse description of the data (i.e., fit after adjustment for model complexity). The best fits were therefore consistent with substantial proliferation in the CD57$^+$ compartments, such that influx from the CD57$^-$ compartments typically contributed only ~5% of all newly generated CD57$^+$ memory T cells (Table 1).

DISCUSSION

In this study, we used *in vivo* deuterium labeling and *ex vivo* analyses of telomere length, telomerase activity, and intracellular

Figure 2. Ki67$^+$ Cells Are Readily Detectable in the CD57$^+$ Memory T Cell Pool

(A) Representative flow cytometric data from a labeled volunteer (DW01) showing cytosolic expression of Ki67 among memory CD4$^+$ (top) or CD8$^+$ T cells (bottom) gated as CD57$^-$ (blue) or CD57$^+$ (red).

(B) Percent cytosolic expression of Ki67 among memory CD4$^+$ (top) or CD8$^+$ T cells (bottom) gated as CD57$^-$ (blue triangles) or CD57$^+$ (red circles). *$p < 0.05$, **$p < 0.01$. Paired samples Wilcoxon test.

(C) Representative flow cytometric data from unlabeled volunteers ($n = 2$) showing cytosolic/nuclear expression of Ki67 among memory CD4$^+$ T cells gated as CD57$^-$ (blue) or CD57$^+$ (red). HC07 was seronegative for CMV.

(D) Top: percent cytosolic/nuclear expression of Ki67 among memory CD4$^+$ T cells gated as CD57$^-$ (blue triangles) or CD57$^+$ (red circles). Bottom: percent expression of CD28 among the corresponding Ki67$^+$CD57$^-$ (blue triangles) and Ki67$^+$CD57$^+$ memory CD4$^+$ T cells (red circles). **$p < 0.01$. Paired samples Wilcoxon test.

(E) Representative flow cytometric data from unlabeled volunteers ($n = 2$) showing cytosolic/nuclear expression of Ki67 among memory CD8$^+$ T cells gated as CD57$^-$ (blue) or CD57$^+$ (red). HC02 was seropositive for CMV, and HC08 was seronegative for CMV.

(F) Top: percent cytosolic/nuclear expression of Ki67 among memory CD8$^+$ T cells gated as CD57$^-$ (blue triangles) or CD57$^+$ (red circles). Bottom: percent expression of CD28 among the corresponding Ki67$^+$CD57$^-$ (blue triangles) and Ki67$^+$CD57$^+$ memory CD8$^+$ T cells (red circles). *$p < 0.05$, **$p < 0.01$. Paired samples Wilcoxon test.

(G) Phenotypic characteristics of Ki67$^+$CD57$^-$ and Ki67$^+$CD57$^+$ memory CD4$^+$ (top) or CD8$^+$ T cells (bottom) shown overlaid on density clouds representing the corresponding total CD4$^+$ (top) or CD8$^+$ T cell populations (bottom). Related to (A).

(H) Phenotypic characteristics of Ki67$^+$CD57$^-$ and Ki67$^+$CD57$^+$ memory CD4$^+$ T cells shown overlaid on density clouds representing the corresponding total CD4$^+$ T cell populations. Related to (C). Key as in (G).

(I) Phenotypic characteristics of Ki67$^+$CD57$^-$ and Ki67$^+$CD57$^+$ memory CD8$^+$ T cells shown overlaid on density clouds representing the corresponding total CD8$^+$ T cell populations. Related to (E). Key as in (G).

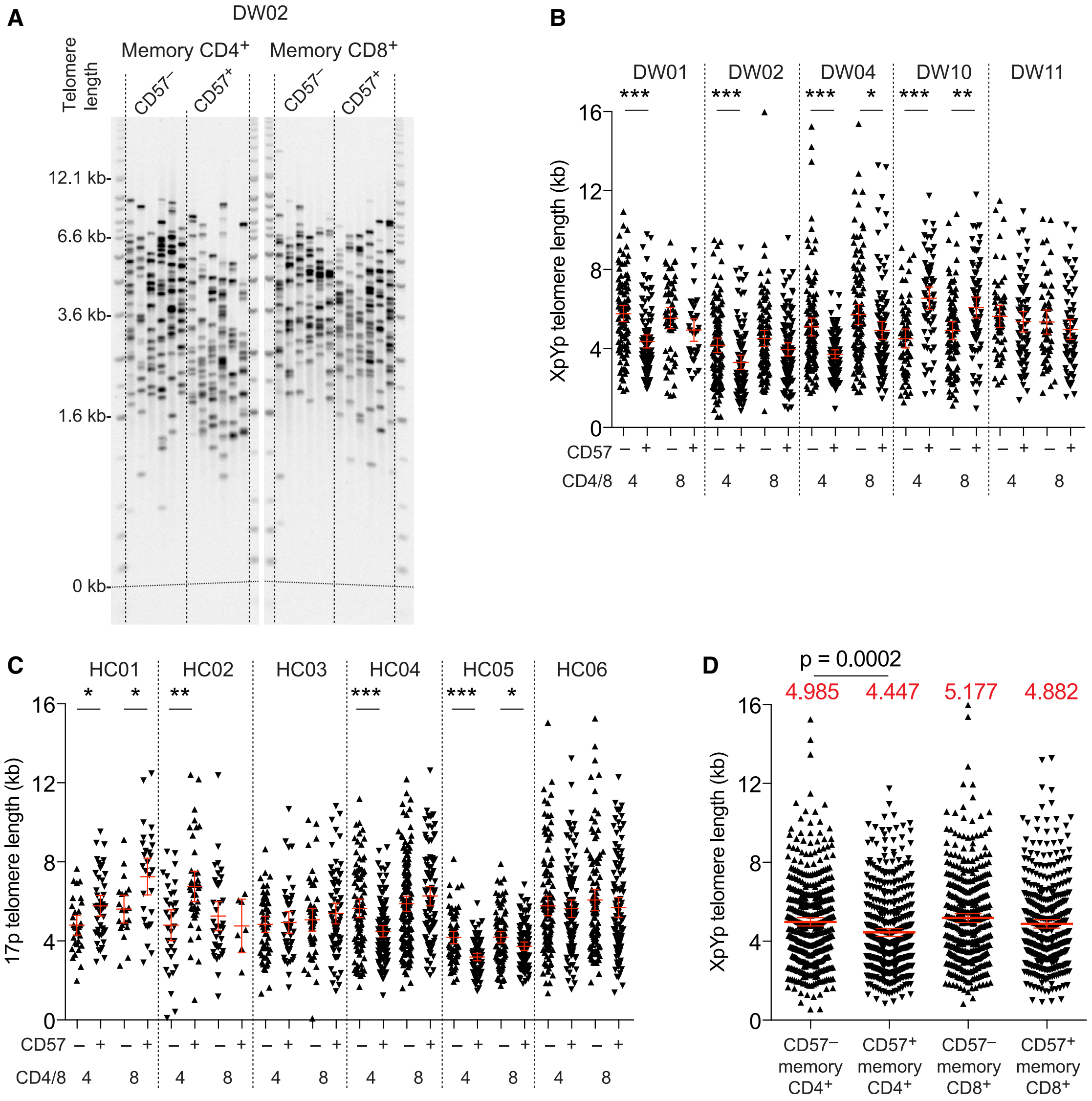

Figure 3. CD57− and CD57+ Memory T Cells Have Similar Division Histories

(A) Representative single telomere length analysis (STELA) data showing XpYp telomere lengths among CD57− and CD57+ memory CD4+ or CD8+ T cells sampled from a labeled volunteer (DW02).

(B) XpYp telomere lengths among CD57− and CD57+ memory CD4+ or CD8+ T cells sampled from labeled volunteers (cohort 2). *$p < 0.05$, **$p < 0.01$, ***$p < 0.001$. Mann-Whitney *U* test.

(C) 17p telomere lengths among CD57− and CD57+ memory CD4+ or CD8+ T cells sampled from unlabeled volunteers (cohort 3). *$p < 0.05$, **$p < 0.01$, ***$p < 0.001$. Mann-Whitney *U* test.

(D) Pooled XpYp telomere length data for the volunteers shown in (B). Red lines show means with 95% confidence intervals. Mean values are specified above each column. Significance was assessed using the Mann-Whitney *U* test.

expression of the cell-cycle marker Ki67 to investigate the paradigm that replicatively senescent memory T cells can be identified via the surrogate marker CD57. We detected similar rates of proliferation among CD57− and CD57+ memory T cells in both coreceptor-defined lineages. These results were supported by flow cytometric analyses, which revealed the presence of actively dividing cells in the corresponding CD57+ memory T cell populations. Marginally higher levels of telomerase activity were detected among CD57− memory T cells compared with CD57+ memory T cells in the CD4+ and CD8+ lineages, consistent with a relatively small biological effect, and in line with recent observations (Fali et al., 2018), telomere lengths were maintained to a slightly greater extent among CD57− memory CD4+ T cells compared with CD57+ memory CD4+ T cells. In contrast, telomere lengths were distributed around similar means in the CD57− and CD57+ memory CD8+ T cell populations. Mathematical modeling of the experimental data further suggested that self-renewal via intracompartmental proliferation rather than

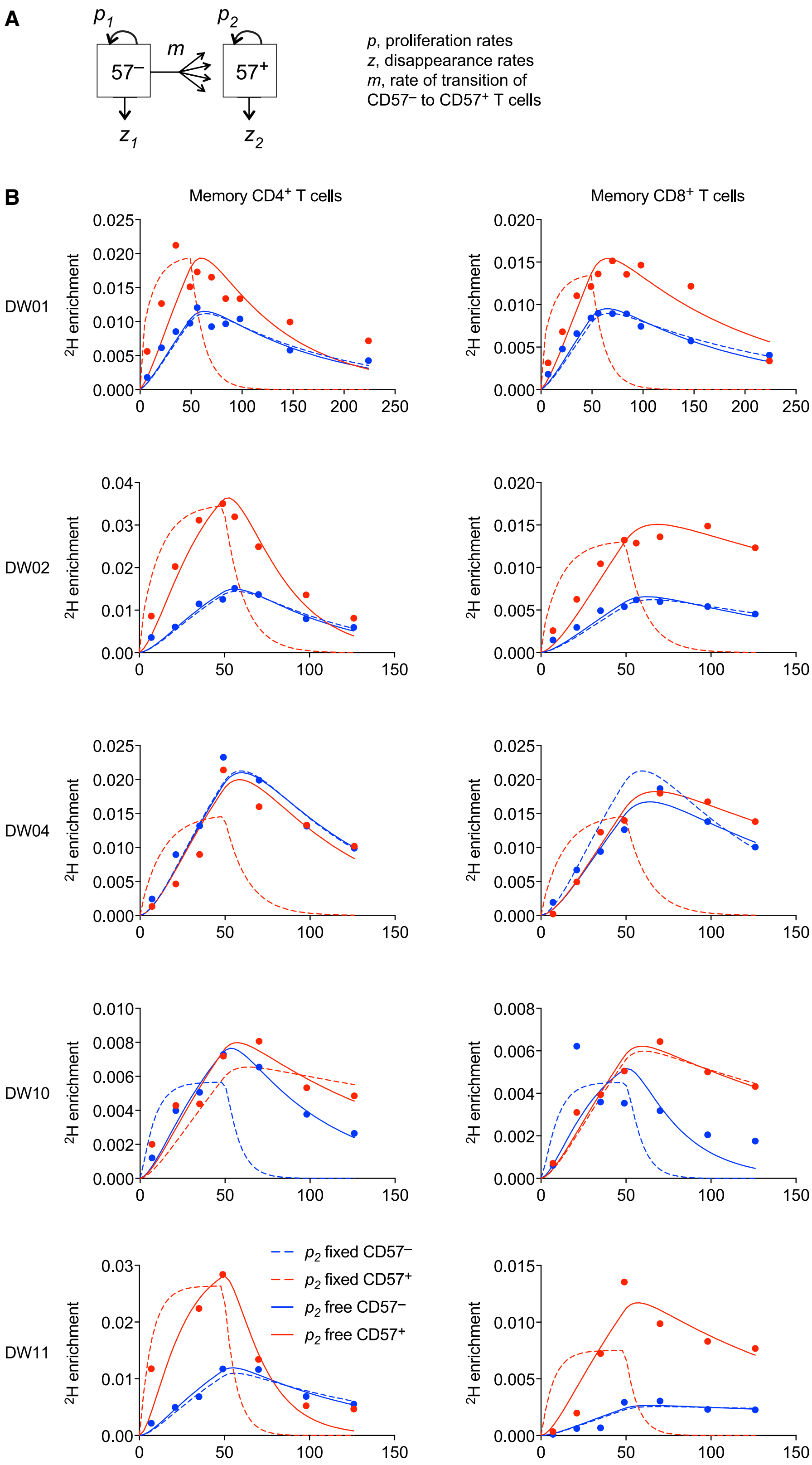

Figure 4. CD57⁻ and CD57⁺ Memory T Cells Self-Renew *In Vivo*

(A) Schematic representation of the mathematical model.

(B) Model fits to the measured data (dots) for CD57⁻ and CD57⁺ memory CD4⁺ (left) or CD8⁺ T cells (right) with p_2 constrained to zero (dashed lines) or free (solid lines).

Table 1. Parameter Estimates from Model Fits to the Experimental Data

ID	p_1 (% per day)	SE (% per day)	z_1 (% per day)	SE (% per day)	p_2 (% per day)	SE (% per day)	z_2 (% per day)	SE (% per day)	mR (% per day)	SE (% per day)	Self-renewal (%)
CD4$^+$ T Cells											
DW01	0.33	0.04	0.0088	0.27	0.59	0.08	1.24	0.37	0.02	0.01	97
DW02	0.65	0.06	1.89	0.31	2.08	0.23	3.77	0.58	0.17	0.03	93
DW04	0.53	N/D	1.43	N/D	0.53	N/D	1.65	N/D	0.00	N/D	100
DW10	0.58	0.05	1.80	0.28	0.49	0.05	0.92	0.22	0.00	0.00	99
DW11	0.44	0.06	1.24	0.33	1.64	0.02	5.24	0.66	0.45	0.02	79
Median	0.53		1.43		0.59		1.65		0.02		97
CD8$^+$ T Cells											
DW01	0.26	0.02	0.73	0.13	0.40	0.03	0.70	0.13	0.02	0.00	96
DW02	0.22	0.02	0.86	0.22	0.42	0.04	0.45	0.19	0.04	0.01	92
DW04	0.37	N/D	0.91	N/D	0.37	N/D	0.62	N/D	0.00	N/D	100
DW10	0.55	0.17	3.60	1.53	0.34	0.11	0.63	0.56	0.02	0.02	95
DW11	0.07	0.01	0.24	0.34	0.32	0.03	0.81	0.04	0.07	0.01	83
Median	0.26		0.86		0.37		0.63		0.02		95

The best-fit estimates are shown. A limited number of data points were available from one volunteer (DW04). The asymptotic covariance matrix method was used to calculate standard errors (SEs). The percentage of new CD57$^+$ T cells generated via intracompartmental proliferation (right column) was calculated as 100 × $p_2/(p_2 + mR)$. ID, identification number; N/D, not determined.

replenishment via phenotypic conversion was by far the most abundant source of newly generated CD57$^+$ memory CD4$^+$ and CD57$^+$ memory CD8$^+$ T cells.

CD57 was originally recognized as a differentiation antigen on the surface of NK cells (Abo and Balch, 1981) and subsequently associated with other lymphocyte subsets in germinal centers (Ritchie et al., 1983). In peripheral blood, CD57$^+$ memory T cells accumulate throughout life, especially after infection with CMV (Gratama et al., 1989). These associations with age and persistent antigenic drive were mechanistically linked in a seminal *in vitro* study, which reported that replicatively senescent memory CD8$^+$ T cells expressed CD57 (Brenchley et al., 2003). However, an earlier study had reached a different conclusion (Izquierdo et al., 1990), and later experiments showed that CD57$^+$ memory CD8$^+$ T cells were able to proliferate *in vitro* in the presence of certain growth factors, potentially mimicking the *in vivo* microenvironment (Chong et al., 2008). Similar findings were reported in another study, although markedly higher response frequencies on a per-cell basis were noted in the CD57$^-$ subset compared with the CD57$^+$ subset (Le Priol et al., 2006). Nonetheless, the proportion of responding cells in the CD57$^+$ subset was more than sufficient to maintain homeostatic turnover, at least according to a deuterium labeling study of bulk memory T cell populations (Zhang et al., 2013).

T_{EMRA} cells are somewhat resistant to apoptosis (Gupta and Gollapudi, 2007) and retain deuterium in the CD8$^+$ lineage with an estimated half-life of approximately 25 years, assuming simple exponential decay without phenotypic conversion (Ladell et al., 2008). In response to extreme stimulation with supraphysiological concentrations of phytohemagglutinin and interleukin-2, CD8$^+$ T_{EMRA} cells that expressed CD57 were recently found to be more susceptible to cell death than CD8$^+$ T_{EMRA} cells that lacked CD57 (Verma et al., 2017). This observation was thought to indicate a functional dichotomy between CD57-defined subsets within the CD8$^+$ T_{EMRA} compartment. However, it does not necessarily follow that a similar dichotomy exists under homeostatic conditions, because terminally differentiated CD57$^+$ memory CD8$^+$ T cells may be protected from excessive stimulation *in vivo* by a lack of costimulatory receptors, such as CD27 and CD28.

In summary, we have shown that CD57$^+$ memory T cells in the CD4$^+$ and CD8$^+$ lineages self-renew *in vivo*, enabling the long-term maintenance of functionally replete immunological memory. It remains to be determined how this process is regulated in terms of antigenic drive versus homeostatic signals as a function of differentiation status, but nonetheless, it is clear from the presented data that replicatively senescent memory T cells cannot be defined solely via surface expression of CD57.

STAR★METHODS

Detailed methods are provided in the online version of this paper and include the following:

- KEY RESOURCES TABLE
- RESOURCE AVAILABILITY
  - Lead Contact
  - Materials Availability
  - Data and Code Availability
- EXPERIMENTAL MODEL AND SUBJECT DETAILS
- METHOD DETAILS
  - Measurement and Analysis of Deuterium Enrichment in T Cell DNA
  - Flow Cytometry and Cell Sorting
  - Single Chromosome Telomere Length Analysis
  - Measurement of Telomerase Activity

CellPress
OPEN ACCESS

- QUANTIFICATION AND STATISTICAL ANALYSIS
  - General Statistics
  - Mathematical Modeling
  - Inclusion of Telomere Length Data in Model Fits
  - Estimation of Telomere Length Change
  - Fitting Procedure

SUPPLEMENTAL INFORMATION

Supplemental Information can be found online at https://doi.org/10.1016/j.celrep.2020.108501.

ACKNOWLEDGMENTS

The authors extend their profound thanks to all study participants. This work was funded by Cancer Research UK (grant C17199/A18246), the Medical Research Council (grant G1001052), and the Wellcome Trust (grant 093053/Z/10/Z). J.L.-B. was supported by the European Union Seventh Framework Programme (grant 317040, QuanTI). Pilot studies were funded in part by the National Institutes of Health (NIH) via grant RO1 AI43866 to M.K.H. and grants R37 AI40312 and U01 AI43641 to J.M.M., who also received a Burroughs Wellcome Fund Clinical Scientist Award in Translational Research and was supported by an NIH Director's Pioneer Award, part of the NIH Roadmap for Medical Research, via grant DPI OD00329. Research conducted at the University of California San Francisco Clinical and Translational Science Institute under the guidance of J.M.M. was supported by the National Center for Research Resources, part of the NIH Roadmap for Medical Research, via grant UL1 RR024131-01. D.A.P. was supported by a Wellcome Trust Senior Investigator Award (100326/Z/12/Z). B.A. was supported by the European Union Seventh Framework Programme (grant 317040, QuanTI), Leukemia and Lymphoma Research (grant 15012), Medical Research Council (grants G1001052 and J007439), and Wellcome Trust via an Investigator Award (103865/Z/14/Z).

AUTHOR CONTRIBUTIONS

M.K.H., J.M.M., D.A.P., D.C.M., and K.L. designed experiments; R.A., K.L.M., R.E.J., L.R., Y.Z., and K.L. performed experiments; C.B., M.K.H., J.M.M., D.M.B., D.A.P., D.C.M., and K.L. supervised experiments; R.A., K.L.M., R.E.J., L.R., D.M.B., D.C.M., and K.L. analyzed data; J.L.-B. and B.A. modeled data; D.A.P., D.C.M., B.A., and K.L. wrote the manuscript. All authors contributed intellectually and approved the manuscript.

DECLARATION OF INTERESTS

The authors declare no competing interests.

Received: April 9, 2020
Revised: August 26, 2020
Accepted: November 18, 2020
Published: December 15, 2020

REFERENCES

Abo, T., and Balch, C.M. (1981). A differentiation antigen of human NK and K cells identified by a monoclonal antibody (HNK-1). J. Immunol. *127*, 1024–1029.

Addo, M.M., Draenert, R., Rathod, A., Verrill, C.L., Davis, B.T., Gandhi, R.T., Robbins, G.K., Basgoz, N.O., Stone, D.R., Cohen, D.E., et al. (2007). Fully differentiated HIV-1 specific CD8$^+$ T effector cells are more frequently detectable in controlled than in progressive HIV-1 infection. PLoS ONE *2*, e321.

Ahmed, R., Westera, L., Drylewicz, J., Elemans, M., Zhang, Y., Kelly, E., Reljic, R., Tesselaar, K., de Boer, R.J., Macallan, D.C., et al. (2015). Reconciling estimates of cell proliferation from stable isotope labeling experiments. PLoS Comput. Biol. *11*, e1004355.

Ahmed, R., Roger, L., Costa Del Amo, P., Miners, K.L., Jones, R.E., Boelen, L., Fali, T., Elemans, M., Zhang, Y., Appay, V., et al. (2016). Human stem cell-like memory T cells are maintained in a state of dynamic flux. Cell Rep. *17*, 2811–2818.

Appay, V., van Lier, R.A., Sallusto, F., and Roederer, M. (2008). Phenotype and function of human T lymphocyte subsets: consensus and issues. Cytometry A *73*, 975–983.

Benito, J.M., Ortiz, M.C., León, A., Sarabia, L.A., Ligos, J.M., Montoya, M., Garcia, M., Ruiz-Mateos, E., Palacios, R., Cabello, A., et al.; ECRIS integrated in the Spanish AIDS Research Network (2018). Class-modeling analysis reveals T-cell homeostasis disturbances involved in loss of immune control in elite controllers. BMC Med. *16*, 30.

Bodnar, A.G., Kim, N.W., Effros, R.B., and Chiu, C.P. (1996). Mechanism of telomerase induction during T cell activation. Exp. Cell Res. *228*, 58–64.

Brenchley, J.M., Karandikar, N.J., Betts, M.R., Ambrozak, D.R., Hill, B.J., Crotty, L.E., Casazza, J.P., Kuruppu, J., Migueles, S.A., Connors, M., et al. (2003). Expression of CD57 defines replicative senescence and antigen-induced apoptotic death of CD8$^+$ T cells. Blood *101*, 2711–2720.

Burnham, K.P., and Anderson, D.R. (2002). Model Selection and Multimodel Inference: A Practical Information-Theoretic Approach, Second Edition (Springer-Verlag).

Busch, R., Neese, R.A., Awada, M., Hayes, G.M., and Hellerstein, M.K. (2007). Measurement of cell proliferation by heavy water labeling. Nat. Protoc. *2*, 3045–3057.

Capper, R., Britt-Compton, B., Tankimanova, M., Rowson, J., Letsolo, B., Man, S., Haughton, M., and Baird, D.M. (2007). The nature of telomere fusion and a definition of the critical telomere length in human cells. Genes Dev. *21*, 2495–2508.

Casazza, J.P., Betts, M.R., Price, D.A., Precopio, M.L., Ruff, L.E., Brenchley, J.M., Hill, B.J., Roederer, M., Douek, D.C., and Koup, R.A. (2006). Acquisition of direct antiviral effector functions by CMV-specific CD4$^+$ T lymphocytes with cellular maturation. J. Exp. Med. *203*, 2865–2877.

Chattopadhyay, P.K., Betts, M.R., Price, D.A., Gostick, E., Horton, H., Roederer, M., and De Rosa, S.C. (2009). The cytolytic enzymes granyzme A, granzyme B, and perforin: expression patterns, cell distribution, and their relationship to cell maturity and bright CD57 expression. J. Leukoc. Biol. *85*, 88–97.

Chong, L.K., Aicheler, R.J., Llewellyn-Lacey, S., Tomasec, P., Brennan, P., and Wang, E.C. (2008). Proliferation and interleukin 5 production by CD8^{hi} CD57$^+$ T cells. Eur. J. Immunol. *38*, 995–1000.

Collins, K. (2006). The biogenesis and regulation of telomerase holoenzymes. Nat. Rev. Mol. Cell Biol. *7*, 484–494.

Costa Del Amo, P., Lahoz-Beneytez, J., Boelen, L., Ahmed, R., Miners, K.L., Zhang, Y., Roger, L., Jones, R.E., Marraco, S.A.F., Speiser, D.E., et al. (2018). Human T_{SCM} cell dynamics in vivo are compatible with long-lived immunological memory and stemness. PLoS Biol. *16*, e2005523.

Day, C.L., Kaufmann, D.E., Kiepiela, P., Brown, J.A., Moodley, E.S., Reddy, S., Mackey, E.W., Miller, J.D., Leslie, A.J., DePierres, C., et al. (2006). PD-1 expression on HIV-specific T cells is associated with T-cell exhaustion and disease progression. Nature *443*, 350–354.

De Boer, R.J., and Noest, A.J. (1998). T cell renewal rates, telomerase, and telomere length shortening. J. Immunol. *160*, 5832–5837.

Fali, T., Fabre-Mersseman, V., Yamamoto, T., Bayard, C., Papagno, L., Fastenackels, S., Zoorab, R., Koup, R.A., Boddaert, J., Sauce, D., and Appay, V. (2018). Elderly human hematopoietic progenitor cells express cellular senescence markers and are more susceptible to pyroptosis. JCI Insight *3*, e95319.

Freeman, G.J., Wherry, E.J., Ahmed, R., and Sharpe, A.H. (2006). Reinvigorating exhausted HIV-specific T cells via PD-1-PD-1 ligand blockade. J. Exp. Med. *203*, 2223–2227.

Gerdes, J., Schwab, U., Lemke, H., and Stein, H. (1983). Production of a mouse monoclonal antibody reactive with a human nuclear antigen associated with cell proliferation. Int. J. Cancer *31*, 13–20.

Gratama, J.W., Fridell, E., Lenkei, R., Oosterveer, M.A., Ljungström, I., Tanke, H.J., and Linde, A. (1989). Correlation between cytomegalovirus and

Toxoplasma gondii serology and lymphocyte phenotypes in peripheral blood and cord blood. Scand. J. Infect. Dis. *21*, 611–616.

Gupta, S., and Gollapudi, S. (2007). Effector memory CD8$^+$ T cells are resistant to apoptosis. Ann. N Y Acad. Sci. *1109*, 145–150.

Hegedus, A., Nyamweya, S., Zhang, Y., Govind, S., Aspinall, R., Mashanova, A., Jansen, V.A., Whittle, H., Jaye, A., Flanagan, K.L., and Macallan, D.C. (2014). Protection versus pathology in aviremic and high viral load HIV-2 infection—the pivotal role of immune activation and T-cell kinetics. J. Infect. Dis. *210*, 752–761.

Hellerstein, M., Hanley, M.B., Cesar, D., Siler, S., Papageorgopoulos, C., Wieder, E., Schmidt, D., Hoh, R., Neese, R., Macallan, D., et al. (1999). Directly measured kinetics of circulating T lymphocytes in normal and HIV-1-infected humans. Nat. Med. *5*, 83–89.

Izquierdo, M., Balboa, M.A., Fernández-Rañada, J.M., Figuera, A., Torres, A., Iriondo, A., and López-Botet, M. (1990). Relation between the increase of circulating CD3$^+$ CD57$^+$ lymphocytes and T cell dysfunction in recipients of bone marrow transplantation. Clin. Exp. Immunol. *82*, 145–150.

Kern, F., Khatamzas, E., Surel, I., Frömmel, C., Reinke, P., Waldrop, S.L., Picker, L.J., and Volk, H.D. (1999). Distribution of human CMV-specific memory T cells among the CD8$^{pos.}$ subsets defined by CD57, CD27, and CD45 isoforms. Eur. J. Immunol. *29*, 2908–2915.

Ladell, K., Hellerstein, M.K., Cesar, D., Busch, R., Boban, D., and McCune, J.M. (2008). Central memory CD8$^+$ T cells appear to have a shorter lifespan and reduced abundance as a function of HIV disease progression. J. Immunol. *180*, 7907–7918.

Lahoz-Beneytez, J., Elemans, M., Zhang, Y., Ahmed, R., Salam, A., Block, M., Niederalt, C., Asquith, B., and Macallan, D. (2016). Human neutrophil kinetics: modeling of stable isotope labeling data supports short blood neutrophil half-lives. Blood *127*, 3431–3438.

Le Priol, Y., Puthier, D., Lécureuil, C., Combadière, C., Debré, P., Nguyen, C., and Combadière, B. (2006). High cytotoxic and specific migratory potencies of senescent CD8$^+$ CD57$^+$ cells in HIV-infected and uninfected individuals. J. Immunol. *177*, 5145–5154.

McCune, J.M., Hanley, M.B., Cesar, D., Halvorsen, R., Hoh, R., Schmidt, D., Wieder, E., Deeks, S., Siler, S., Neese, R., and Hellerstein, M. (2000). Factors influencing T-cell turnover in HIV-1-seropositive patients. J. Clin. Invest. *105*, R1–R8.

Miller, I., Min, M., Yang, C., Tian, C., Gookin, S., Carter, D., and Spencer, S.L. (2018). Ki67 is a graded rather than a binary marker of proliferation versus quiescence. Cell Rep. *24*, 1105–1112.e5.

Neese, R.A., Siler, S.Q., Cesar, D., Antelo, F., Lee, D., Misell, L., Patel, K., Tehrani, S., Shah, P., and Hellerstein, M.K. (2001). Advances in the stable isotope-mass spectrometric measurement of DNA synthesis and cell proliferation. Anal. Biochem. *298*, 189–195.

Nociari, M.M., Telford, W., and Russo, C. (1999). Postthymic development of CD28$^-$CD8$^+$ T cell subset: age-associated expansion and shift from memory to naive phenotype. J. Immunol. *162*, 3327–3335.

Northfield, J.W., Loo, C.P., Barbour, J.D., Spotts, G., Hecht, F.M., Klenerman, P., Nixon, D.F., and Michaëlsson, J. (2007). Human immunodeficiency virus type 1 (HIV-1)-specific CD8$^+$ T_{EMRA} cells in early infection are linked to control of HIV-1 viremia and predict the subsequent viral load set point. J. Virol. *81*, 5759–5765.

Patel, A.A., Zhang, Y., Fullerton, J.N., Boelen, L., Rongvaux, A., Maini, A.A., Bigley, V., Flavell, R.A., Gilroy, D.W., Asquith, B., et al. (2017). The fate and lifespan of human monocyte subsets in steady state and systemic inflammation. J. Exp. Med. *214*, 1913–1923.

Petrovas, C., Casazza, J.P., Brenchley, J.M., Price, D.A., Gostick, E., Adams, W.C., Precopio, M.L., Schacker, T., Roederer, M., Douek, D.C., and Koup, R.A. (2006). PD-1 is a regulator of virus-specific CD8$^+$ T cell survival in HIV infection. J. Exp. Med. *203*, 2281–2292.

Petrovas, C., Chaon, B., Ambrozak, D.R., Price, D.A., Melenhorst, J.J., Hill, B.J., Geldmacher, C., Casazza, J.P., Chattopadhyay, P.K., Roederer, M., et al. (2009). Differential association of programmed death-1 and CD57 with ex vivo survival of CD8$^+$ T cells in HIV infection. J. Immunol. *183*, 1120–1132.

Previs, S.F., Hazey, J.W., Diraison, F., Beylot, M., David, F., and Brunengraber, H. (1996). Assay of the deuterium enrichment of water via acetylene. J. Mass Spectrom. *31*, 639–642.

Ritchie, A.W., James, K., and Micklem, H.S. (1983). The distribution and possible significance of cells identified in human lymphoid tissue by the monoclonal antibody HNK-1. Clin. Exp. Immunol. *51*, 439–447.

Soetaert, K., and Petzoldt, T. (2010). Inverse modelling, sensitivity and Monte Carlo analysis in R using package FME. J. Stat. Softw. *33*, 1–28.

Takata, H., and Takiguchi, M. (2006). Three memory subsets of human CD8$^+$ T cells differently expressing three cytolytic effector molecules. J. Immunol. *177*, 4330–4340.

Trautmann, L., Janbazian, L., Chomont, N., Said, E.A., Gimmig, S., Bessette, B., Boulassel, M.R., Delwart, E., Sepulveda, H., Balderas, R.S., et al. (2006). Upregulation of PD-1 expression on HIV-specific CD8$^+$ T cells leads to reversible immune dysfunction. Nat. Med. *12*, 1198–1202.

van Leeuwen, E.M., Gamadia, L.E., Baars, P.A., Remmerswaal, E.B., ten Berge, I.J., and van Lier, R.A. (2002). Proliferation requirements of cytomegalovirus-specific, effector-type human CD8$^+$ T cells. J. Immunol. *169*, 5838–5843.

Verma, K., Ogonek, J., Varanasi, P.R., Luther, S., Bünting, I., Thomay, K., Behrens, Y.L., Mischak-Weissinger, E., and Hambach, L. (2017). Human CD8$^+$ CD57$^-$ T_{EMRA} cells: too young to be called "old". PLoS ONE *12*, e0177405.

Vrisekoop, N., den Braber, I., de Boer, A.B., Ruiter, A.F., Ackermans, M.T., van der Crabben, S.N., Schrijver, E.H., Spierenburg, G., Sauerwein, H.P., Hazenberg, M.D., et al. (2008). Sparse production but preferential incorporation of recently produced naive T cells in the human peripheral pool. Proc. Natl. Acad. Sci. U. S. A. *105*, 6115–6120.

Vrisekoop, N., Drylewicz, J., Van Gent, R., Mugwagwa, T., Van Lelyveld, S.F., Veel, E., Otto, S.A., Ackermans, M.T., Vermeulen, J.N., Huidekoper, H.H., et al. (2015). Quantification of naive and memory T-cell turnover during HIV-1 infection. AIDS *29*, 2071–2080.

Wege, H., Chui, M.S., Le, H.T., Tran, J.M., and Zern, M.A. (2003). SYBR Green real-time telomeric repeat amplification protocol for the rapid quantification of telomerase activity. Nucleic Acids Res. *31*, e3.

Westera, L., Zhang, Y., Tesselaar, K., Borghans, J.A., and Macallan, D.C. (2013). Quantitating lymphocyte homeostasis in vivo in humans using stable isotope tracers. Methods Mol. Biol. *979*, 107–131.

Zhang, Y., de Lara, C., Worth, A., Hegedus, A., Laamanen, K., Beverley, P., and Macallan, D. (2013). Accelerated in vivo proliferation of memory phenotype CD4$^+$ T-cells in human HIV-1 infection irrespective of viral chemokine co-receptor tropism. PLoS Pathog. *9*, e1003310.

STAR★METHODS

KEY RESOURCES TABLE

REAGENT or RESOURCE	SOURCE	IDENTIFIER
Antibodies		
Anti-CD3–APC-H7 (clone SK7)	BD Biosciences	Cat#641415; RRID:AB_2870309
Anti-CD4–PE-Cy5.5 (clone S3.5)	Thermo Fisher Scientific	Cat#MHCD0418; RRID:AB_10376013
Anti-CD8–BV711 (clone RPA-T8)	BioLegend	Cat#301044; RRID:AB_2562906
Anti-CD14–V500 (clone M5E2)	BD Biosciences	Cat#561391; RRID:AB_10611856
Anti-CD19–V500 (clone HIB19)	BD Biosciences	Cat#561121; RRID:AB_10562391
Anti-CD27–QD605 (clone CLB-27/1)	Thermo Fisher Scientific	Cat#Q10065; RRID:AB_2556450
Anti-CD28–APC (clone CD28.2)	BD Biosciences	Cat#559770; RRID:AB_398666
Anti-CD28–BV421 (clone CD28.2)	BioLegend	Cat#302930; RRID:AB_2561910
Anti-CD45RA–ECD (clone 2H4LDH11LDB9)	Beckman Coulter	Cat#M2711U; RRID:AB_10640553
Anti-CD45RA–PE (clone HI100)	BD Biosciences	Cat#555489; RRID:AB_395880
Anti-CD45RO–ECD (clone UCHL1)	Beckman Coulter	Cat#IM2712U; RRID:AB_10639537
Anti-CD57–FITC (clone NK-1)	BD Biosciences	Cat#555619; RRID:AB_395986
Anti-CD57–PE-Cy7 (clone NK-1)	BioLegend	Cat#359624; RRID:AB_2632689
Anti-CD127–BV421 (clone A019D5)	BioLegend	Cat#351310; RRID:AB_10960140
Anti-CCR7–BV421 (clone G043H7)	BioLegend	Cat#353208; RRID:AB_11203894
Anti-CCR7–FITC (clone 150503)	BD Biosciences	Cat#561271; RRID:AB_10561679
Anti-CCR7–PE-Cy7 (clone 3D12)	BD Biosciences	Cat#557648; RRID:AB_396765
Anti-CXCR3–BV421 (clone G025H7)	BioLegend	Cat#353716; RRID:AB_2561448
Anti-Ki67–AF647 (clone B56)	BD Biosciences	Cat#558615; RRID:AB_647130
Anti-Ki67–FITC (clone B56)	BD Biosciences	Cat#556026; RRID:AB_396302
Anti-PD-1–BV421 (clone EH12.2H7)	BioLegend	Cat#329920; RRID:AB_10960742
Biological Samples		
Peripheral blood from adults infected with HIV-1	San Francisco General Hospital, San Francisco, CA, USA	N/A
Peripheral blood from healthy adult volunteers	St George's Hospital, London, UK	N/A
Peripheral blood from healthy adult volunteers	Cardiff University School of Medicine, Cardiff, UK	N/A
Chemicals, Peptides, and Recombinant Proteins		
Heavy water (^2H_2O)	Cambridge Isotope Laboratories	Cat#DLM-4TPB-PK
Pentafluorobenzyl hydroxylamine	Sigma-Aldrich	Cat#194484
Sodium dodecyl sulfate	Thermo Fisher Scientific	Cat#10593355
Tris(hydroxymethyl) methylamine	Thermo Fisher Scientific	Cat#77-86-1
Ethylenediaminetetraacetic acid	Sigma-Aldrich	Cat#E6511-100G
Sodium hydroxide	Thermo Fisher Scientific	Cat#J/7620/15
Sodium phosphate dibasic	Sigma-Aldrich	Cat#7558-79-4
Hydrochloric acid	Thermo Fisher Scientific	Cat#7647-01-0
Critical Commercial Assays		
LIVE/DEAD Fixable Aqua Dead Cell Stain Kit	Thermo Fisher Scientific	Cat#L34966
Cytofix/Cytoperm Kit	BD Biosciences	Cat#554715
Foxp3 Transcription Factor Staining Buffer Kit	Thermo Fisher Scientific	Cat#00-5521-00
QIAmp DNA Mini Kit	QIAGEN	Cat#51304
QIAmp DNA Micro Kit	QIAGEN	Cat#56304

(*Continued on next page*)

Continued		
REAGENT or RESOURCE	SOURCE	IDENTIFIER
Oligonucleotides		
XpYpE2: TTGTCTCAGGGTCCTAGTG	Eurofins Genomics	Custom
17pserev1: GAATCCACGGATTGCTTTGTGTAC	Eurofins Genomics	Custom
Telorette2: TGCTCCGTGCATCTGGCATCTAACCCT	Eurofins Genomics	Custom
Teltail: TGCTCCGTGCATCTGGCATC	Eurofins Genomics	Custom
Software and Algorithms		
DiVa version 8	BD Biosciences	https://www.bdbiosciences.com/en-us
FlowJo software version 9.9.4	FlowJo LLC	https://www.flowjo.com
Phoretix 1D Quantifier	Nonlinear Dynamics	http://www.nonlinear.com/about/totallab
Prism version 8	GraphPad	https://www.graphpad.com
Other		
DreamTaq polymerase	Thermo Fisher Scientific	Cat#EP0702
Pwo polymerase	Sigma-Aldrich	Cat#11644955001
dNTPs	Promega	Cat#U1511
α-^{33}P dCTP	PerkinElmer	Cat#BLU013H100UC
Megaprime	VWR	Cat#RPN1607
Hybond-XL	VWR	Cat#RPN15205
Agarose MP	Sigma-Aldrich	Cat#11388983001
1 kb ladder	Agilent	Cat#201115
2.5 kb ladder	Bio-Rad	Cat#1708205
FACSVantage SE	BD Biosciences	https://www.bdbiosciences.com/en-us
FACSAria	BD Biosciences	https://www.bdbiosciences.com/en-us
Special Order Research Product FACSAria II	BD Biosciences	https://www.bdbiosciences.com/en-us
GC/MS (5873/6980)	Agilent	https://www.agilent.com
DB-17 column	Agilent	https://www.agilent.com
Tetrad2 Thermal Cycler	Bio-Rad	https://www.bio-rad.com
Typhoon FLA 9500 Phosphorimager	GE Healthcare	https://www.cytivalifesciences.com/

RESOURCE AVAILABILITY

Lead Contact

Further information and requests for reagents and resources should be directed to and will be fulfilled by the Lead Contact, Kristin Ladell (ladellk@gmail.com).

Materials Availability

This study did not generate new unique reagents.

Data and Code Availability

The datasets reported in this study are available on request from the Lead Contact, Kristin Ladell (ladellk@gmail.com).

EXPERIMENTAL MODEL AND SUBJECT DETAILS

Three groups of human volunteers participated in this work. Cohort 1: volunteers with chronic HIV-1 infection (aged 36–53 years) were recruited for preliminary *in vivo* labeling studies (n = 4 males; Table S1). All were antiretroviral drug-free and seropositive for CMV. Cohort 2: healthy volunteers (aged 29–83 years) were recruited for more extensive *in vivo* labeling studies (n = 3 females; n = 5 males; Table S1). All were seronegative for hepatitis C virus and HIV-1 and seropositive for CMV. Cohort 3: additional healthy volunteers (aged 28–58 years) were recruited for phenotypic studies and measurements of telomere length and telomerase activity (n = 7 females; n = 7 males). Similar experiments were performed using venous blood samples donated by 5 of the 8 volunteers in cohort 2. All studies were conducted in accordance with the principles of the Declaration of Helsinki. Ethical approval was granted by the University of California Committee on Human Research (cohort 1), the London-

Chelsea Research Ethics Committee (cohort 2), and the Cardiff University School of Medicine Research Ethics Committee (cohort 3).

METHOD DETAILS

Measurement and Analysis of Deuterium Enrichment in T Cell DNA

T cell proliferation *in vivo* was measured using deuterium (^2H) labeling as described previously (Busch et al., 2007; Hellerstein et al., 1999; Ladell et al., 2008; McCune et al., 2000; Neese et al., 2001; Westera et al., 2013). Briefly, volunteers received heavy water (^2H_2O) orally for 7 weeks (Figure 1A), and deuterium incorporation into the DNA of flow-sorted T cells was quantified via gas chromatography/mass spectrometry (Agilent 5873/6980) (Ladell et al., 2008). DNA was released by boiling and hydrolyzed according to standard protocols, and deoxyribonucleosides were derivatized using pentafluorobenzyl hydroxylamine (Sigma-Aldrich). Gas chromatography/mass spectrometry was performed in negative chemical ionization mode using a DB-17 column (Agilent). The M+1/M+0 isotopomer ratio was monitored at mass-to-charge (*m/z*) 436/435. To normalize for body water enrichment, weekly saliva samples were analyzed for ^2H_2O content via calcium carbide-induced acetylene generation, monitoring at *m/z* 27/26 (Previs et al., 1996).

Flow Cytometry and Cell Sorting

T cell subsets of interest were flow-sorted from freshly isolated peripheral blood mononuclear cells (PBMCs) at >98% purity using a FACSVantage SE, a FACSAria, or a Special Order Research Product FACSAria II (all from BD Biosciences). Cells were stained with combinations of the following reagents: (1) anti-CD3–APC-H7 (clone SK7), anti-CD14–V500 (clone M5E2), anti-CD19–V500 (clone HIB19), anti-CD28–APC (clone CD28.2), anti-CD45RA–PE (clone HI100), anti-CD57–FITC (clone NK-1), anti-CCR7–FITC (clone 150503), and anti-CCR7–PE-Cy7 (clone 3D12) from BD Biosciences; (2) anti-CD4–PE-Cy5.5 (clone S3.5), anti-CD27–QD605 (clone CLB-27/1), and LIVE/DEAD Fixable Aqua from Thermo Fisher Scientific; (3) anti-CD8–BV711 (clone RPA-T8), anti-CD28–BV421 (clone CD28.2), anti-CD57–PE-Cy7 (clone NK-1), anti-CD127–BV421 (clone A019D5), anti-CCR7–BV421 (clone G043H7), anti-CXCR3–BV421 (clone G025H7), and anti-PD-1–BV421 (clone EH12.2H7) from BioLegend; and (4) anti-CD45RA–ECD (clone 2H4LDH11LDB9) and anti-CD45RO–ECD (clone UCHL1) from Beckman Coulter. Viable $CD57^-$ and $CD57^+$ memory T cells were identified in the $CD4^+$ and/or $CD8^+$ lineages after exclusion of $CD27^{bright}CD45RO^-$ (Figure 1C) or $CD45RA^+CCR7^+$ events (Figure S1). Cytosolic expression of Ki67 was evaluated using anti-Ki67–AF647 (clone B56; BD Biosciences) in conjunction with a Cytofix/Cytoperm Kit (BD Biosciences), and cytosolic/intranuclear expression of Ki67 was evaluated using anti-Ki67–FITC (clone B56; BD Biosciences) in conjunction with a Foxp3 Transcription Factor Staining Buffer Kit (Thermo Fisher Scientific). Data were analyzed with FlowJo software version 9.9.4 (FlowJo LLC).

Single Chromosome Telomere Length Analysis

DNA was extracted from 3,000 flow-sorted T cells using a QIAmp DNA Micro Kit (QIAGEN). Single telomere length analysis (STELA) was carried out at the XpYp or the 17p telomere as described previously (Capper et al., 2007). Briefly, 0.75 μL of the Telorette-2 linker (10 μM) was added to genomic DNA eluted in 35 μL of Tris (10 mM). Multiple PCRs were then performed for each test DNA. Each reaction was set up in a final volume of 10 μL containing 250 pg of DNA and the telomere-adjacent and Teltail primers at a final concentration of 0.5 μM in 75 mM Tris-HCl pH 8.8, 20 mM $(NH_4)_2SO_4$, 0.01% Tween-20, and 1.5 mM $MgCl_2$, with 0.5 U of a 10:1 mixture of Taq (Thermo Fisher Scientific) and Pwo polymerase (Sigma-Aldrich). The reactions were processed in a Tetrad2 Thermal Cycler (Bio-Rad). DNA fragments were resolved via 0.5% Tris-acetate-EDTA agarose gel electrophoresis and identified via Southern hybridization with a random-primed α-^{33}P-labeled (PerkinElmer) TTAGGG repeat probe, together with probes specific for molecular weight markers at 1 kb (Agilent) and 2.5 kb (Bio-Rad). Hybridized fragments were detected using a Typhoon FLA 9500 Phosphorimager (GE Healthcare). The molecular weights of the DNA fragments were calculated using Phoretix 1D Quantifier (Nonlinear Dynamics).

Measurement of Telomerase Activity

Flow-sorted T cells were lyzed and assayed in two steps using a modified SYBR Green real-time quantitative telomeric repeat amplification protocol (Wege et al., 2003). Standard curves were obtained from serial dilutions of a 293T cell extract with known telomerase activity. Experimental telomerase activity was calculated with reference to 293T cells and expressed as relative telomerase activity (Ct_{293T}/Ct_{sample}).

QUANTIFICATION AND STATISTICAL ANALYSIS

General Statistics

Unmatched groups were compared using the Mann-Whitney *U* test, and matched groups were compared using the paired samples Wilcoxon test. Significance was assigned at $p < 0.05$.

Mathematical Modeling

Mechanistic ordinary differential equation-based models were developed to assess the dynamics of $CD57^-$ and $CD57^+$ memory T cells (Costa Del Amo et al., 2018; Patel et al., 2017). These subsets were modeled as dependent populations to investigate the

possibility that label acquisition in the CD57$^+$ compartment was a consequence of proliferation-linked differentiation in the CD57$^-$ compartment. Accordingly, CD57$^-$ and CD57$^+$ memory T cells were allowed to proliferate and die or exit the circulation, and CD57$^-$ memory T cells were allowed to gain expression of CD57. The phenotypic conversion of CD57$^-$ memory T cells into CD57$^+$ memory T cells was considered over n rounds of division, including the possibility that $n = 0$.

In the applied model, CD57$^-$ memory T cells (x_1) became CD57$^+$ memory T cells (x_2) at a rate m:

$$\frac{dx_1}{dt} = p_1x_1 - z_1x_1$$

$$\frac{dx_2}{dt} = p_2x_2 - z_2x_2 + mx_1$$

where p_1 and p_2 are the rates of proliferation of CD57$^-$ and CD57$^+$ memory T cells, respectively, and z_1 and z_2 are the rates of disappearance of CD57$^-$ and CD57$^+$ memory T cells, respectively. The possibility that surface expression of CD57 could be acquired during clonal expansion was accommodated in the permitted values for m (bounds during fitting [0,40]).

To minimize the number of free parameters, label enrichment among CD57$^+$ memory T cells was assumed to originate either from dividing CD57$^-$ memory T cells that differentiated into CD57$^+$ memory T cells or from dividing CD57$^+$ memory T cells. A model in which the acquisition of CD57 was not coincident with clonal expansion resulted in a substantially worse fit to the data and was not pursued further. The fraction of label thus became:

$$\frac{dL_1}{dt} = p_1b_wU_t - z_1^*L_1$$

$$\frac{dL_2}{dt} = (p_2 + mR)b_wU_t - z_2^*L_2$$

where p_1 and p_2 are as above, z_1* and z_2* are the rates of loss of labeled CD57$^-$ and CD57$^+$ memory T cells, respectively, L_1 and L_2 are the fractions of labeled deoxyadenosine among CD57$^-$ and CD57$^+$ memory T cells, respectively, R is the ratio of CD57$^-$ to CD57$^+$ memory T cells (x_1/x_2), and b_w is the amplification factor estimated from label acquisition among granulocytes, assuming 100% turnover in 7 weeks. Data were available from 4 volunteers and gave a population average value for b_w of 3.5, consistent with previous studies (Ahmed et al., 2015; Lahoz-Beneytez et al., 2016). The value of b_w was therefore fixed at 3.5. Finally, $U(t)$ is an empirical function used to describe the availability of label in body water:

$$U(t) = f\left(1 - e^{-\delta t}\right) + \beta e^{-\delta t} \quad \text{during labeling } t \le \tau$$

$$U(t) = f\left(1 - e^{-\delta t}\right) + \beta e^{-\delta t} \quad \text{during labeling } t \le \tau$$

as described previously (Vrisekoop et al., 2008), where $U(t)$ represents the fraction of labeled precursor in body water at time t (in days), f is the fraction of labeled precursor in ingested water, τ is the length of the labeling period, δ is the turnover rate of body water per day, and β is the plasma enrichment attained at the end of day 0. Parameters were estimated by fitting the above functions to the deuterium labeling data measured in saliva. The resulting fits of $U(t)$ to saliva measurements are shown in Figure S2.

Inclusion of Telomere Length Data in Model Fits

The impact of cell division on telomere length was modeled as described previously (De Boer and Noest, 1998). Telomere length data were available from 5 of the 8 volunteers in cohort 2. The model was fitted simultaneously to the labeling data and the telomere length data using the free parameters p_1 and p_2 to describe the rates of proliferation of CD57$^-$ and CD57$^+$ memory T cells, respectively, z_1* and z_2* to describe the rates of disappearance of labeled CD57$^-$ and CD57$^+$ memory T cells, respectively, and mR, the rate of conversion from CD57$^-$ to CD57$^+$ memory T cells (m) multiplied by the ratio of the frequency of CD57$^-$ memory T cells to the frequency of CD57$^+$ memory T cells (R).

Estimation of Telomere Length Change

The rate of change in telomere loss indices for CD57$^-$ memory T cells was defined according to a previous report (De Boer and Noest, 1998) as follows:

$$\frac{d\mu_1}{dt} = 2p_1$$

$$\frac{d\mu_2}{dt} = 2p_2 - mR(\mu_2 - \mu_1 - K)$$

where p_1, p_2, m, and R are as above, K is the length of telomere loss upon clonal expansion in units of division, and μ_1 and μ_2 are the average number of divisions undergone by CD57$^-$ and CD57$^+$ memory T cells, respectively. The difference in telomere length between CD57$^-$ and CD57$^+$ memory T cells was estimated as:

$$\Delta = (\mu_2 - \mu_1)\varepsilon$$

where ε is the average number of base pairs (bp) lost per division (taken to be 50 bp [De Boer and Noest, 1998]), giving the following expression for the difference in telomere length:

$$\Delta_C = \varepsilon\left(\frac{2(p_2 - p_1)}{mR} + K\right)$$

Fitting Procedure

The function $U(t)$ was fitted to the deuterium labeling data measured in saliva, and the free parameters f, β, and δ were estimated for each individual. The resulting parameterized $U(t)$ functions were then used as fixed inputs during simultaneous fitting of the deuterium labeling and telomere length data from CD57$^-$ and CD57$^+$ memory T cells using the equations for L_1, L_2, and Δ_C above. The free parameters were p_1, p_2, z_1, z_2, and mR. As telomerase is highly active during clonal expansion, the telomere length loss index (K) was initially set to 0 (Bodnar et al., 1996; Collins, 2006). This assumption was subsequently relaxed to explore the impact of variations in K (Figure S6). The contribution of self-renewal to the production of new CD57$^+$ memory T cells was defined as:

$$\text{contribution from self-renewal} = \frac{p_2 x_2}{p_2 x_2 + m x_1}$$

$$= \frac{p_2}{p_2 + mR}$$

To ensure that the labeling data and the telomere length data contributed equally to the fit, all residuals were normalized by the mean, and the deuterium residuals were divided by the number of labeling data points. Conclusions were analyzed for robustness against changes in the number of telomere base pairs lost per division. Scenarios in which CD57$^+$ memory T cells did not proliferate were also tested by fixing p_2 to 0. Model performance was evaluated using the F test and the AICc (Burnham and Anderson, 2002). The model was fitted to the data using non-linear least-squares regression implemented via the algorithm Pseudo in the FME package in R (Soetaert and Petzoldt, 2010).

Cell Reports, Volume *33*

Supplemental Information

CD57$^+$ Memory T Cells Proliferate *In Vivo*

Raya Ahmed, Kelly L. Miners, Julio Lahoz-Beneytez, Rhiannon E. Jones, Laureline Roger, Christina Baboonian, Yan Zhang, Eddie C.Y. Wang, Marc K. Hellerstein, Joseph M. McCune, Duncan M. Baird, David A. Price, Derek C. Macallan, Becca Asquith, and Kristin Ladell

SUPPLEMENTAL MATERIAL

1. SUPPLEMENTAL FIGURES

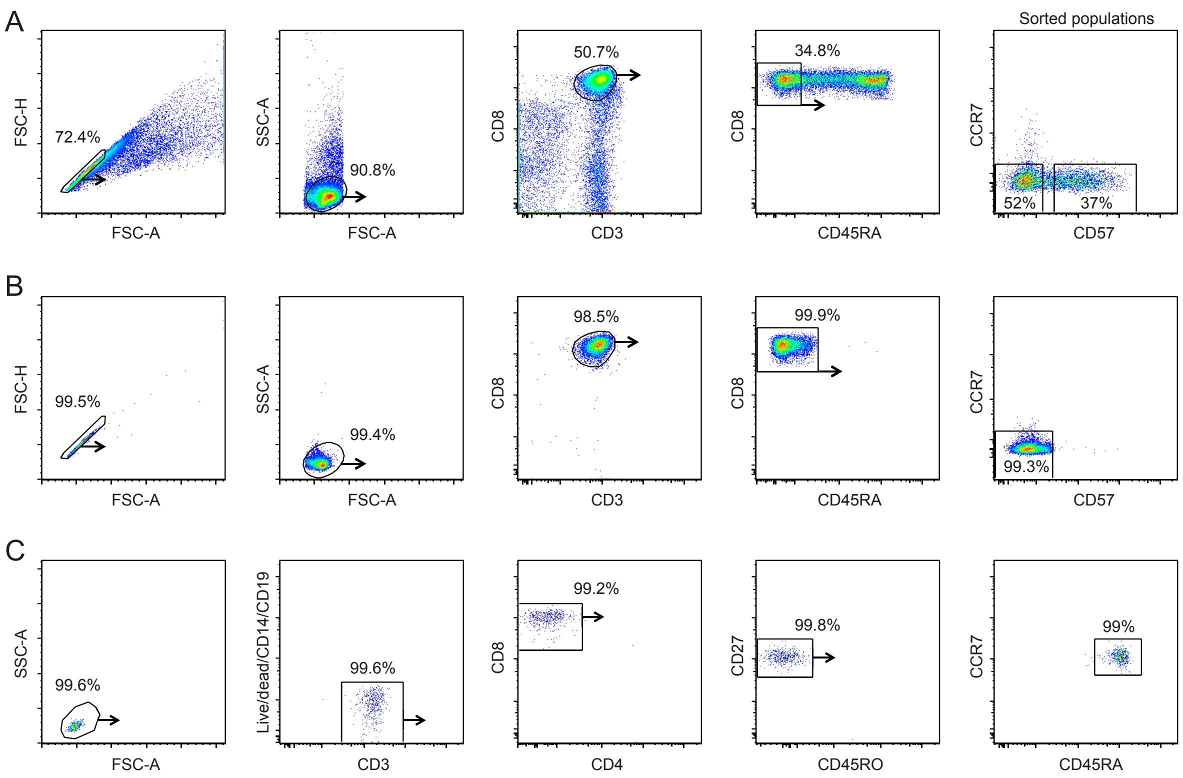

Figure S1. Flow cytometric gating strategy for the pilot ^2H_2O labeling study (Cohort 1) and representative sort purity checks (Cohorts 1 and 2). (A) Successive panels depict the flow cytometric gating strategy used to sort $CD57^-$ and $CD57^+$ memory $CD8^+$ T cells (Cohort 1). Single cells were identified in a forward scatter-area versus forward scatter-height plot, and lymphocytes were identified in a forward scatter-area versus side scatter-area plot. Sort gates were then fixed on $CD3^+CD8^+CD45RA^-CCR7^-CD57^-$ and $CD3^+CD8^+CD45RA^-CCR7^-CD57^+$ memory T cells. (B) Representative sort purity check for $CD3^+CD8^+CD45RA^-CCR7^-CD57^-$ memory T cells (Cohort 1). (C) Representative sort purity check for $CD3^+CD8^+CD27^+CD45RO^-CCR7^+$ naive T cells (Cohort 2). This abundant subset was analyzed to preserve $CD57^-$ and $CD57^+$ memory T cells. Related to Figure 1.

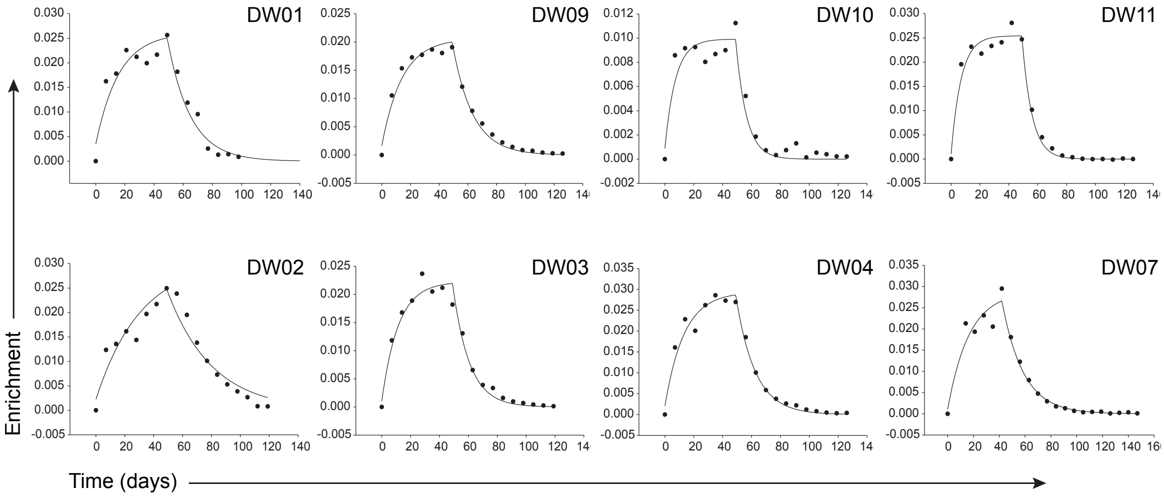

Figure S2. ^{2}H enrichment in body water. Measured ^{2}H enrichments in body water (dots) with best-fit curves (solid lines) for all volunteers in Cohort 2. Young volunteers are shown on the top row, and elderly volunteers are shown on the bottom row. Related to Figure 1.

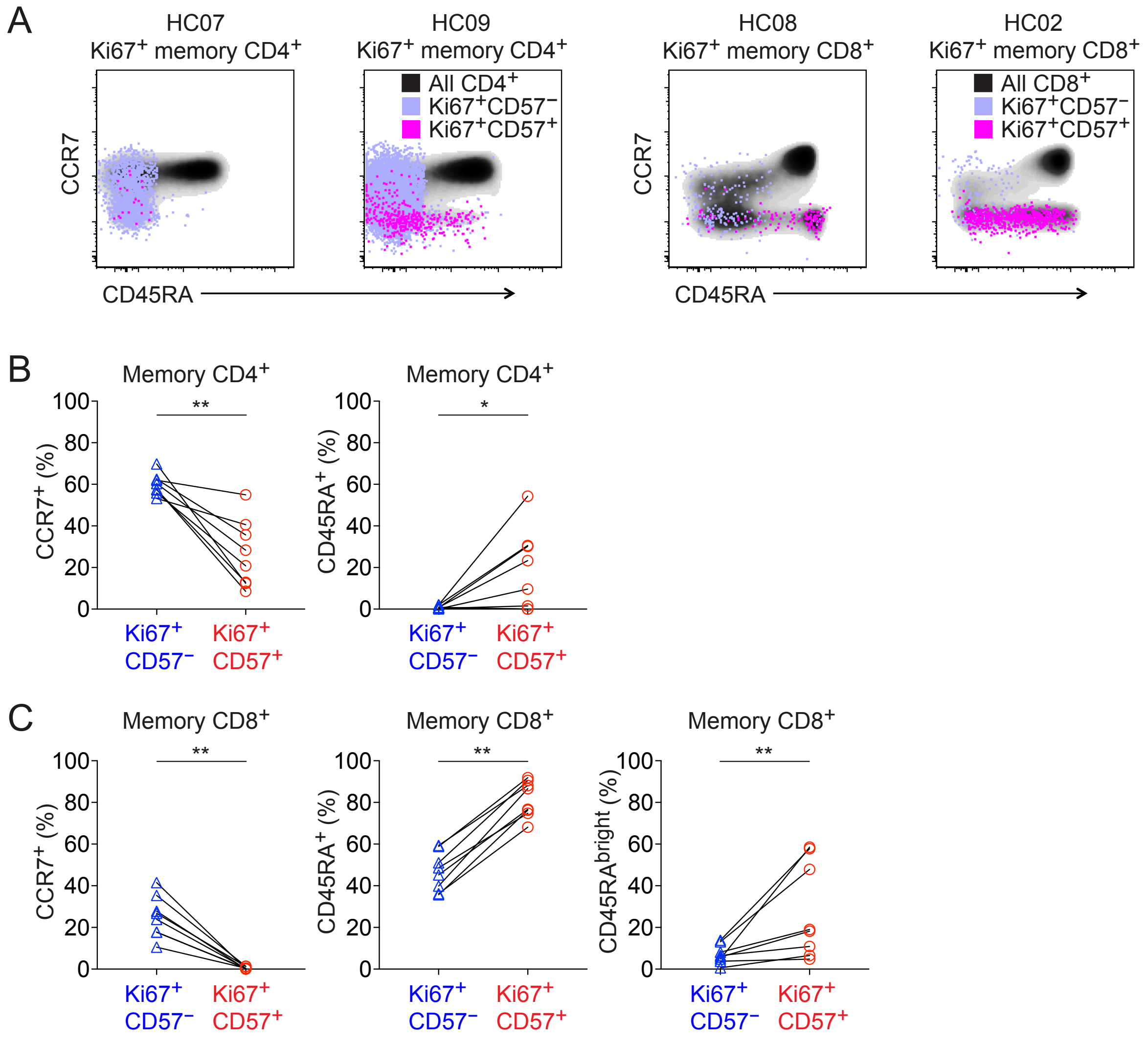

Figure S3. Expression of Ki67 among CD57$^-$ and CD57$^+$ memory T cells. (A) Representative flow cytometric data from unlabeled volunteers (n = 4) showing the phenotypic characteristics of Ki67$^+$CD57$^-$ and Ki67$^+$CD57$^+$ memory CD4$^+$ (left panels) or CD8$^+$ T cells (right panels) overlaid on density clouds representing the corresponding total CD4$^+$ (left panels) or CD8$^+$ T cell populations (right panels). HC02 was seropositive for CMV. HC07 and HC08 were seronegative for CMV. (B) Percent cytosolic/nuclear expression of CCR7 (left) and CD45RA (right) among Ki67$^+$CD57$^-$ (blue triangles) and Ki67$^+$CD57$^+$ memory CD4$^+$ T cells (red circles). (C) Percent cytosolic/nuclear expression of CCR7 (left) and CD45RA (center and right) among Ki67$^+$CD57$^-$ (blue triangles) and Ki67$^+$CD57$^+$ memory CD8$^+$ T cells (red circles). The right graph shows only CD45RA^{bright} events. *$p < 0.05$, **$p < 0.01$. Paired samples Wilcoxon test. Related to Figure 2.

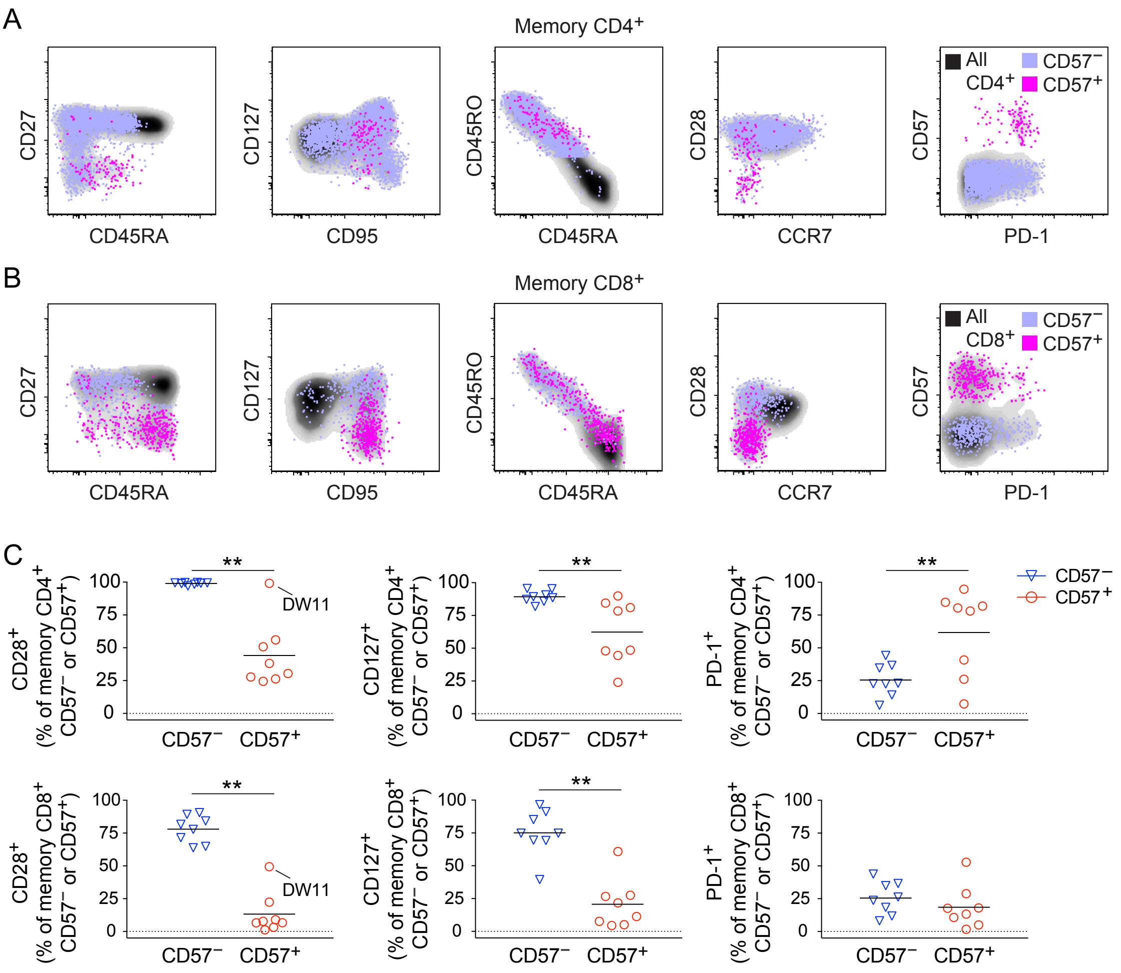

Figure S4. CD57$^+$ memory T cells are phenotypically distinct in the CD4$^+$ and CD8$^+$ lineages. (A) Phenotypic characteristics of CD57$^-$ and CD57$^+$ memory CD4$^+$ T cells from a labeled volunteer (DW01) shown overlaid on density clouds representing the corresponding total CD4$^+$ T cell population. (B) Phenotypic characteristics of CD57$^-$ and CD57$^+$ memory CD8$^+$ T cells from a labeled volunteer (DW01) shown overlaid on density clouds representing the corresponding total CD8$^+$ T cell population. (C) Percent expression of CD28 (left), CD127 (center), and PD-1 (right) among CD57$^-$ and CD57$^+$ memory CD4$^+$ (top) or CD8$^+$ T cells (bottom) sampled from all volunteers in Cohort 2. Unusually high frequencies of CD57$^+$ memory CD4$^+$ and CD57$^+$ memory CD8$^+$ T cells expressed CD28 in one volunteer (DW11). **$p < 0.01$. Paired samples Wilcoxon test. Related to Figure 2.

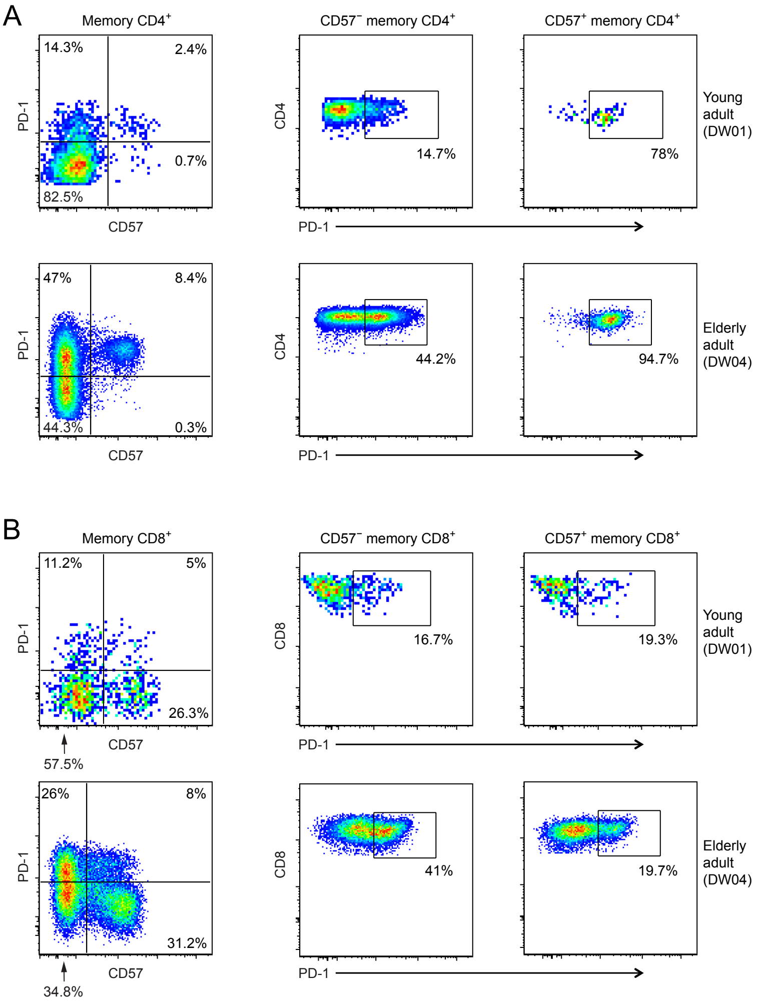

Figure S5. Expression of PD-1 among CD57⁻ and CD57⁺ memory T cells. (A) Representative flow cytometric data showing the expression of PD-1 among CD57$^-$ and CD57$^+$ memory CD4$^+$ T cells. A young volunteer is shown on the top row (DW01). An elderly volunteer is shown on the bottom row (DW04). (B) Representative flow cytometric data showing the expression of PD-1 among CD57$^-$ and CD57$^+$ memory CD8$^+$ T cells. A young volunteer is shown on the top row (DW01). An elderly volunteer is shown on the bottom row (DW04). Related to Figure 2.

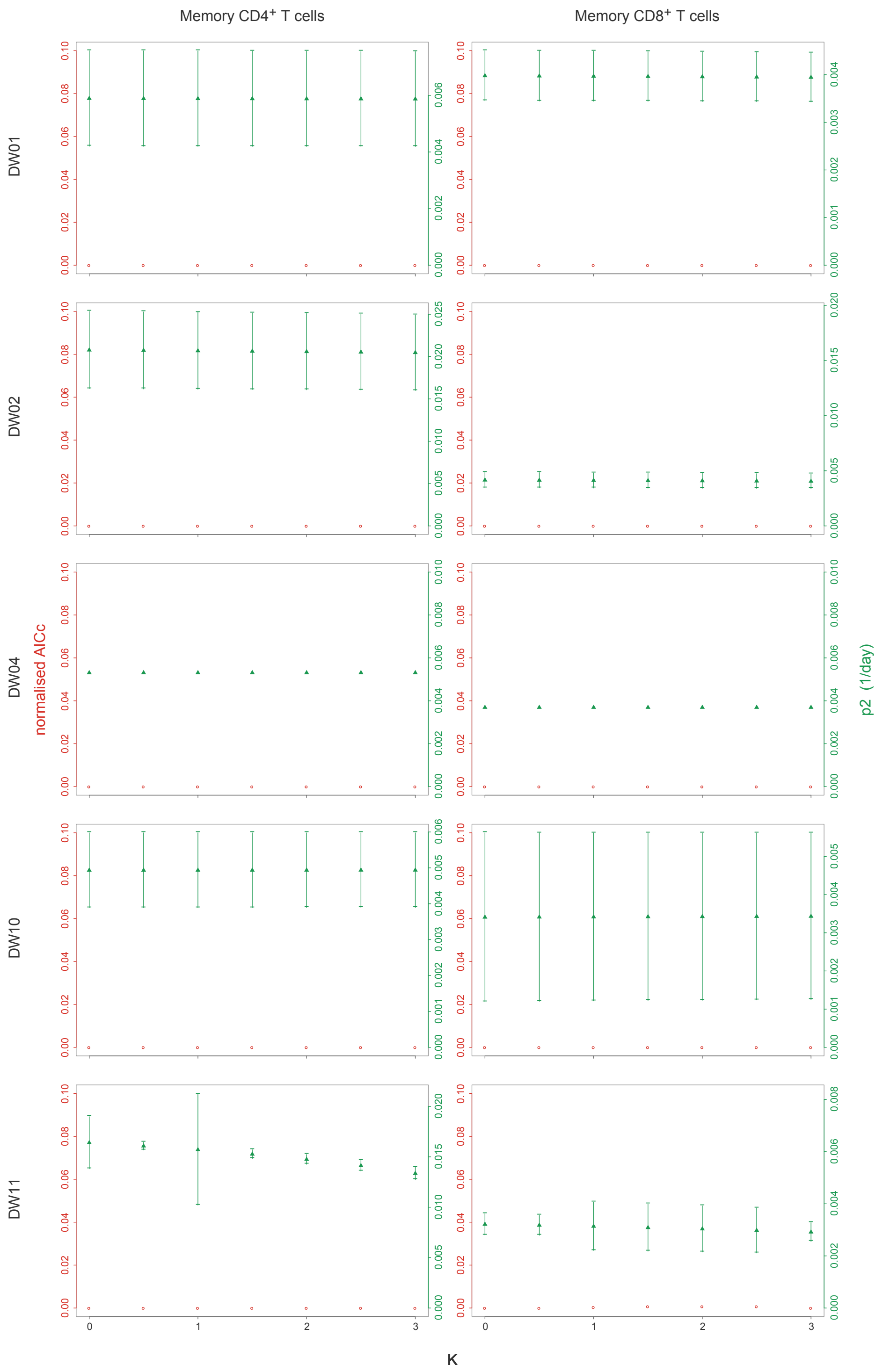

Figure S6. Sensitivity of parameter estimates and AICc values to changes in the telomere loss index shift (*K*). The telomere loss index shift, defined as the length of telomere lost per unit of division, is shown on the x-axes (denoted as *K*). Each plot shows the impact of varying *K* on the normalized AICc (red circles) and the estimated rate of proliferation in the corresponding $CD57^{+}$ memory T cell population (green triangles). Error bars show 95% confidence intervals. Normalized AICc = AICc − AICc of the winning model. Related to Figure 4.

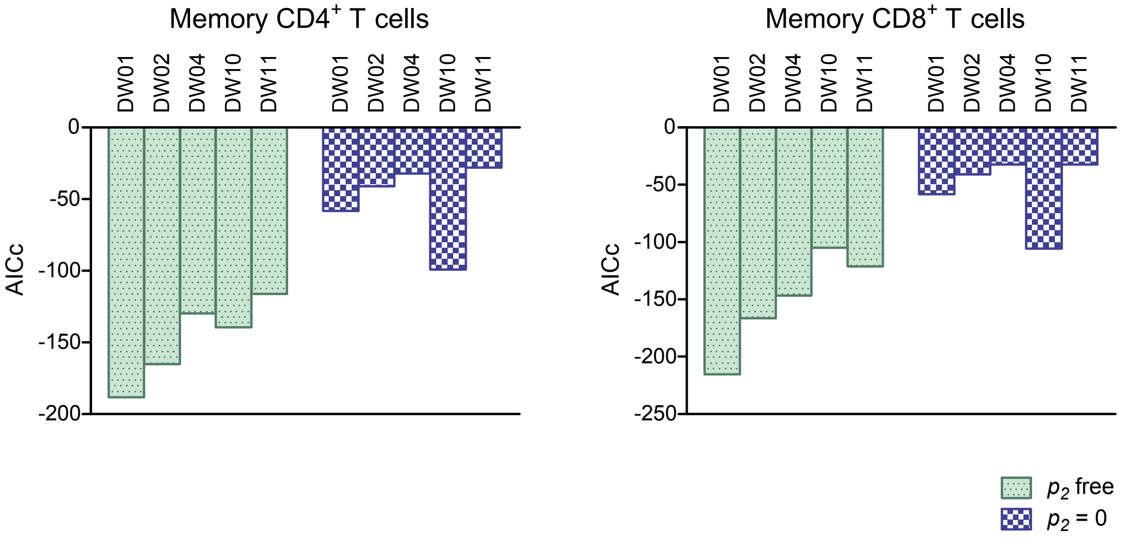

Figure S7. Quality of model fits to the experimental data with p_2 free or constrained to zero. Quality of fit was assessed using the AICc. Lower values indicate a better quality of fit, and higher values indicate a worse quality of fit. The proliferation rate in each $CD57^+$ memory T cell population (p_2) was either free (green) or constrained to zero (blue). Related to Figure 4.

2. SUPPLEMENTAL TABLES

ID	Age	Gender	HIV-1 load (copies/mL plasma)	CD4$^+$ T cell count (x10^9/L)	CD8$^+$ T cell count (x10^9/L)	CMV serology	HIV-1 serology
HI16	53	Male	74,199	0.330	1.397	+	+
HI17	40	Male	2,059	0.852	3.388	+	+
HI19	36	Male	448,343	0.349	2.445	+	+
HI20	38	Male	30,851	0.431	1.646	+	+
DW01	32	Female	NA	0.809	0.460	+	−
DW02	64	Male	NA	0.681	0.314	+	−
DW03	78	Male	NA	0.869	0.405	+	−
DW04	83	Male	NA	0.885	0.579	+	−
DW07	60	Female	NA	0.654	0.241	+	−
DW09	47	Male	NA	1.077	0.800	+	−
DW10	34	Male	NA	0.488	0.977	+	−
DW11	29	Female	NA	1.380	0.864	+	−

Table S1. Clinical details and demographics of HIV-1-infected (HI) and healthy volunteers (DW) included in the labeling studies. NA, not applicable. Related to Figures 1–4.
